# Dopamine signaling reprograms macrophage FAO to alleviate acute lung injury by inhibiting NETosis via the CXCL10-CXCR3 axis

**DOI:** 10.1186/s10020-026-01549-7

**Published:** 2026-07-04

**Authors:** Ximing Liao, Changwen Deng, Jing Gao, Ziqi Yang, Tian Li, Jialin Yao, Muyun Wang, Fengyang Xie, Zhaoqi Li, Kun Wang, Qiang Li, Di Wu, Wujian Xu, Wei Gao

**Affiliations:** 1 Department of Pulmonary and Critical Care Medicine, Shanghai East Hospital, School of Medicine, Tongji University, Shanghai, 200092 China; 2 Department of General Practice, Shanghai Jing’an District Caojiadu Community Health Center, Shanghai, 200040 China; 3 School of Medicine, Saint George’s University, True blue GGD 000, West Indies, Grenada

**Keywords:** Dopamine, Acute lung injury, Macrophage metabolism, Mitochondrial homeostasis, Neutrophil extracellular traps

## Abstract

**Background:**

Dysregulated innate immunity and oxidative stress drive the pathogenesis of acute lung injury/acute respiratory distress syndrome (ALI/ARDS), yet master endogenous regulators that orchestrate inflammation resolution remain elusive.

**Methods:**

This study employed public database mining, clinical data investigation, murine disease models, mouse bone marrow-derived macrophages and neutrophils, and human macrophages from healthy donors and ARDS patients, to investigate the dynamic changes of the dopaminergic signaling system in the acute pulmonary inflammatory environment and its role in regulating macrophage metabolism and neutrophil extracellular trap formation (NETosis).

**Results:**

Public database mining and experimental data reveal accelerated dopamine (DA) turnover during ALI. DA, signaling via D1-like receptors, reprograms macrophage metabolism by enhancing carnitine palmitoyltransferase 1 A (CPT1A)-dependent fatty acid oxidation (FAO) and mitochondrial fitness, which is coupled with the suppression of MAPK/NF-κB and NLRP3 inflammasome activation. These modulated macrophages restrain neutrophilic inflammation by secreting IL-10 to inhibit the CXCL10-CXCR3 axis, thereby curtailing neutrophil hyperactivation and pathogenic NETosis. Crucially, this protective mechanism is conserved in human macrophages from both healthy donors and ARDS patients.

**Conclusion:**

Our findings establish DA as a therapeutic target for recalibrating innate immunity in ALI, providing a mechanistically grounded framework for targeting dopaminergic signaling to resolve dysregulated inflammation, with exploratory preclinical translational implications for ALI/ARDS therapy.

**Graphical abstract:**

**Figure:**
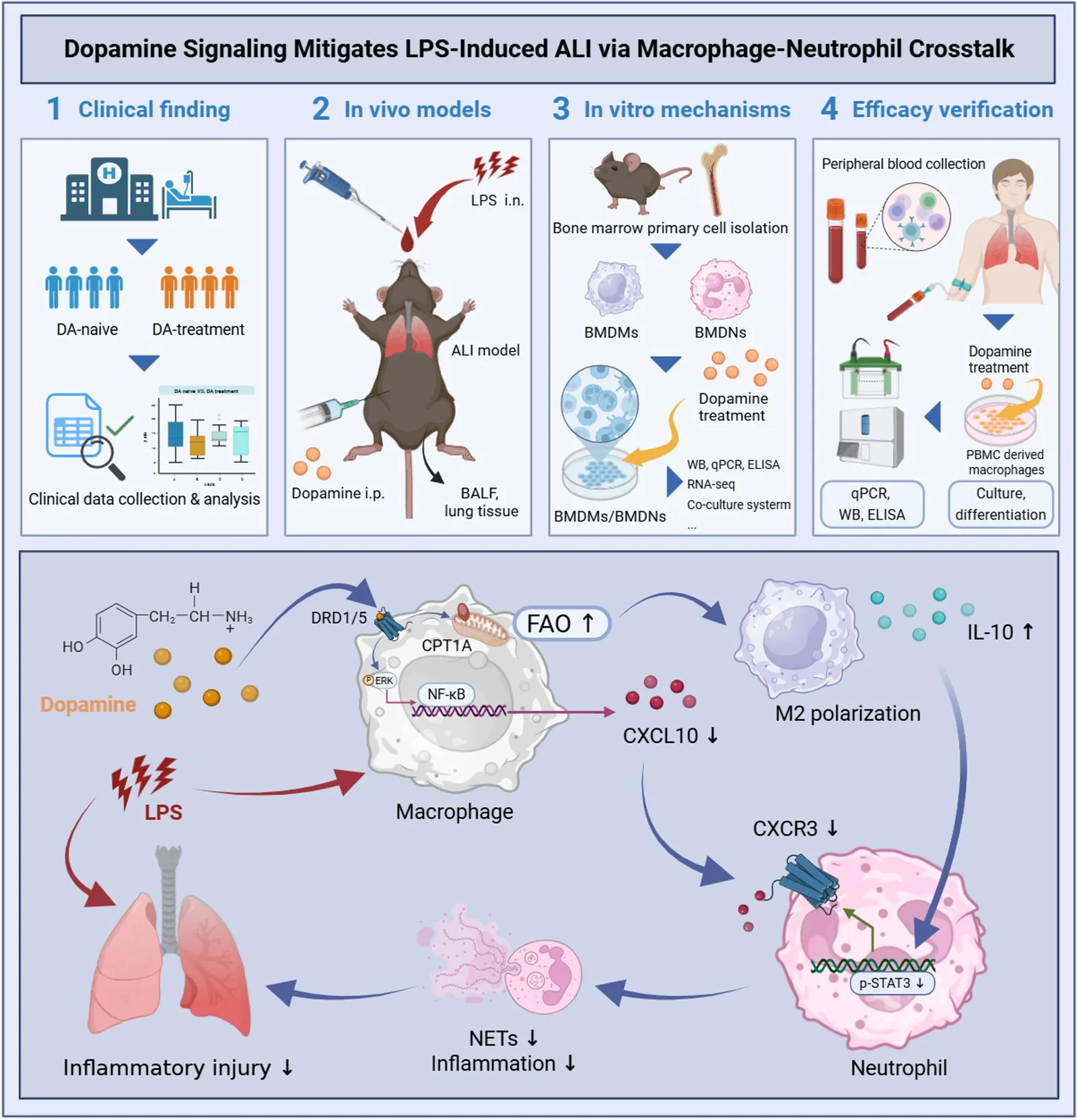
The study design and the mechanism by which DA signaling acting on D1-like receptors modulates macrophage-neutrophil crosstalk via metabolism reprogramming and the CXCL10-CXCR3 axis in ALI/ARDS.

**Supplementary Information:**

The online version contains supplementary material available at https://doi.org/10.1186/s10020-026-01549-7.

## Introduction

Dopamine (DA) has been intensively studied for its crucial roles in motor control, cognition, learning, and reward mechanisms since its initial chemical synthesis in 1910, and particularly after its identification as a key neurotransmitter in the 1950s(Padilla-Coreano and Martínez-Rivera 2025). DA exerts its effects primarily through DA receptors (DRs), which belong to the G protein-coupled receptor superfamily. These receptors are classified into two families: D1-like receptors (DRD1 and DRD5) that stimulate cAMP production, and D2-like receptors (DRD2, DRD3, and DRD4) that inhibit it(Mittal et al. 2017). While historically research on DA signaling has concentrated on the central nervous system, recent studies have expanded its scope to peripheral regulation, including roles in gastrointestinal(Serio and Zizzo 2023) and pulmonary diseases(Ciarka et al. 2007). This expansion aligns with the recognized bidirectional crosstalk between the nervous and immune systems, where DA actively participates by modulating immune cell functions and inflammatory responses. Notably, most immune cells express all DR subtypes and are responsive to DA. For example, DRD2 antagonists could inhibit macrophage-mediated hepatic fibrosis(Qing et al. 2022), whereas its agonists suppressed M1 macrophage polarization in lipopolysaccharide (LPS)-induced pancreatitis model(Han et al. 2020). Our previous work further demonstrated the anti-inflammatory effect of the D1-like receptor agonist SKF38393 in THP-1 macrophages(Meng et al. 2023). Despite these advances, the regulation of immune cell metabolic reprogramming by DA remains an area that has not been thoroughly investigated.

Acute lung injury (ALI)/acute respiratory distress syndrome (ARDS) is driven by uncontrolled pulmonary inflammation and alveolar-capillary barrier disruption, with macrophages serving as central regulators of disease occurrence and progression. During ALI/ARDS, macrophages undergo dynamic polarization: early pro-inflammatory M1 polarization exacerbates tissue injury, whereas subsequent anti-inflammatory M2 polarization facilitates tissue repair(Mosser and Edwards 2008, Wang et al. 2023). This phenotypic switch is closely coupled to metabolic reprogramming, M1 macrophages primarily utilize aerobic glycolysis, while M2 macrophages depend on enhanced mitochondrial oxidative phosphorylation (OXPHOS) and fatty acid oxidation (FAO)(Kelly and O’Neill 2015, Pearce and Pearce 2013). Notably, FAO is a critical driver of M2 polarization, and its rate-limiting enzyme, carnitine palmitoyltransferase 1 A (CPT1A), directly determines polarization efficiency(Wang et al. 2021). Yet, the role of CPT1A-mediated FAO in DA-regulated macrophage polarization during ALI remains unexplored.

In addition to macrophage, neutrophil represents another pivotal effector cell population in ALI, which plays a dual role. Their phagocytic function and formation of neutrophil extracellular traps (NETs) are essential for host defense, yet excessive NETs release exacerbates lung injury by damaging the alveolar barrier and fueling cytokine storms(Laforge et al. 2020). Although NET-targeted therapies, such as neutrophil elastase (NE) inhibitor, show clinical benefits, they act downstream and do not address the root causes of inflammatory amplification(Saffarzadeh 2018). A hallmark of the pathophysiological complexity of ALI is that immune cells do not act in isolation; rather, the crosstalk between macrophages and neutrophils constitutes a key regulatory node within the inflammatory cascade. This interaction is largely mediated by chemokine/receptor axes, such as the C-X-C motif chemokine ligand (CXCL)-C-X-C motif chemokine receptor (CXCR) pathway, which is known to drive neutrophil recruitment and activation in various inflammatory settings(Tokunaga et al. 2018, Lu et al. 2024, Kaur and Singh 2013). Targeting such upstream intercellular communication pathways, which modulate the initiation of inflammation, may therefore offer superior therapeutic potential compared to directly inhibiting downstream effectors like NETs. However, how neuro-immune signaling regulates this specific cell-cell crosstalk in ALI remains poorly understood and warrants comprehensive investigation.

The direct effects of DA on neutrophils remain elusive, with the existing literature being sparse and inconsistent. Only a handful of studies have investigated this interaction, reporting modulatory roles in apoptosis, phagocytosis, and endothelial adhesion(Sookhai et al. 2000, Sookhai et al. 1999). A single recent study further demonstrated that DA suppresses basal neutrophil chemotaxis via D1-like receptors(Marino et al. 2022). Given this limited evidence, DA appears to exert either minimal direct effects on neutrophils or to regulate their function through more complex, indirect pathways. To address these gaps, we hypothesized that DA alleviates ALI via a “D1-like receptor-macrophage-neutrophil” regulatory axis involving metabolic reprogramming and intercellular signaling. Employing murine ALI models, in vitro studies, and human peripheral blood mononuclear cells (PBMCs) validation, this work delineates how DA confers protection by reprogramming macrophage-neutrophil axis. First, DA signaling through D1-like receptor inhibits the pro-inflammatory mitogen-activated protein kinase (MAPK)/nuclear factor kappa-B (NF-κB) signaling and NOD-like receptor family pyrin domain containing 3 (NLRP3) inflammasome activation. Simultaneously, it enhances mitochondrial FAO in a CPT1A-dependent manner to promote an M2 anti-inflammatory phenotype. Consequently, these reprogrammed macrophages suppress neutrophil hyperactivation and NETosis (neutrophil extracellular trap formation) by modulating the CXCL10-CXCR3 axis via interleukin (IL)-10/signal transducer and activator of transcription 3 (STAT3) signaling. Therefore, this study not only underscores the translational potential of targeting dopaminergic signaling as a novel therapeutic avenue but also identifies several promising therapeutic targets for ALI.

## Methods

### Ethical approval

All animal experiments adhered to the ARRIVE guidelines and the Guidelines for the Care and Use of Laboratory Animals issued by the Shanghai Laboratory Animal Management Committee, and were approved by the Institutional Animal Care and Use Committee of the Laboratory Animal Research Center, Tongji University (Permit No. TJBB0372310, Shanghai, China). All procedures were performed under sodium pentobarbital anesthesia, with every effort made to minimize suffering in the animals.

The collection of peripheral blood samples and clinical data from patients with ARDS and healthy volunteers was conducted in accordance with the Declaration of Helsinki and approved by the Ethics Committee of Shanghai East Hospital (Approval No. 2019YYS138, Shanghai, China). All participants provided written informed consent prior to study enrollment.

### Establishment of ALI mouse model with different interventions

The C57BL/6 mice (6–8 weeks old, male) and B6;129S*Cpt1a*^tm1(flox)Smoc^ mice (Cre^−^*Cpt1a*^fl/fl^ and Cre^+^*Cpt1a*^fl/fl^) (6–8 weeks old, both male and female) were obtained from Shanghai Model Organisms Center, Inc (Shanghai, China). The strategy for constructing macrophage conditional *Cpt1a* knockout mice was detailed in our prior work(Wang et al. 2024). Mice were maintained in a specific pathogen-free environment under a 12-hour light-dark cycle, with unrestricted access to standard chow and sterile drinking water. ALI model was induced via intranasal instillation of LPS (10 mg/kg; Sigma-Aldrich, USA) or sterile phosphate-buffered saline (PBS, controls) under intraperitoneal sodium pentobarbital anesthesia (10 mg/mL). Mice were sacrificed at 0, 2, 6, 12, and 24 h, or 4 and 7 days post-challenge for subsequent analyses. Each experimental group contained 5–6 biological replicates, and drug intervention protocols were as follows: DA (300 µg per mouse intraperitoneally; Sigma-Aldrich, USA)(Vohra et al. 2012, Cao et al. 2023), AMG487 (5 mg/kg intraperitoneally; MCE, USA) or recombinant mouse CXCL10 (10 µg per mouse intranasally; PeproTech, USA) was administered 2 h prior to LPS stimulation, with SCH23390 (SCH, 0.5 mg/kg intraperitoneally; MCE, USA) or deoxyribonuclease I (DNase I) (10 mg/kg; intraperitoneally; Roche, Switzerland) given 3 h in advance. To neutralize IL-10, mice were intraperitoneally injected with IL-10 neutralizing antibody (αIL-10) or its isotype control for three consecutive days prior to LPS challenge, at a dose of 350 µg per mouse once daily (both antibodies from BioXcell, USA). Besides, Ly6G neutralizing antibody (αLy6G) or isotype control antibody (300 µg per mouse intraperitoneally; BioXcell, USA) were given 26 h before LPS inhalation, whereas clodronate liposome (Clo-lip) or PBS liposome (PBS-lip) (5 mg/mL, 75 µL per mouse intratracheally; Liposoma BV, the Netherlands) was given 74 h prior to LPS exposure. Randomization and blinding were employed to minimize bias.

### Validation of the mouse model

Following anesthesia administration, bronchoalveolar lavage (BAL) was performed via tracheal instillation and subsequent retrieval of 0.8 mL ice-cold PBS, with a recovery rate of more than 70%. Cells in the BAL fluid (BALF) were pelleted by centrifugation at 220 × g for 5 min at 4 °C. Total cells were counted by trypan blue exclusion. Differential counts were performed on Wright-Giemsa-stained slides, and at least 200 cells were counted per field at 400× magnification. The resulting supernatants were harvested and stored at -80 °C for subsequent analysis. The cytokine concentrations in the BALF supernatant were quantitatively determined using enzyme-linked immunosorbent assay (ELISA) kits. Specifically, the cytokines tested included CXCL1/keratinocyte-derived chemokine (KC, EK296), interleukin-6 (IL-6, EK206), CXCL10 (EK268), all of which were purchased from Multisciences Biotech Co., Ltd., China, as well as DA (Abcam, ab285238, UK).

In another set of parallel experiments, the main lobe of the left lung was harvested from mice without undergoing BAL procedure and fixed in 4% paraformaldehyde, and paraffin-embedded sections were subjected to hematoxylin and eosin (H&E) staining for histological evaluation. Lung injury severity was blindly scored by two independent researchers using a validated scoring system, which assesses the following parameters(Matute-Bello et al. 2011): (1) intra-alveolar neutrophil infiltration, (2) interstitial neutrophil infiltration, (3) hyaline membrane formation, (4) intra-alveolar proteinaceous exudates, (5) alveolar wall thickening (each parameter scored 0–2). The total histological injury score was calculated as: [20 × (1) + 14 × (2) + 7 × (3) + 7 × (4) + 2 × (5)] / (number of high-power fields ×100), normalized to a continuous scale of 0–1. For the assessment of neutrophil accumulation and activation status, slides were subjected to immunostaining with primary anti-Ly6G antibody (Abcam, ab25377, 1:200, UK), MPO (Servicebio, GB11224, 1:500, China), Cit-H3 (Zenbio, R24572, 1:200, China), NE (Abcam, ab68672, 1:200, UK) and a subsequent incubation step with FITC-labeled goat anti-rat IgG secondary antibody (Servicebio, GB22302, 1:100, China) or Cy3-labeled goat anti-rabbit IgG secondary antibody (Servicebio, GB21303, 1:300, China). The nuclei were stained with 4′,6-diamidino-2-phenylindole (DAPI). Images were captured on the Nikon Eclipse C1 microscope (Nikon, Japan). For the evaluation of in vivo NETs accumulation in lung tissues, the numbers of Cit-H3/NE double-positive foci were manually enumerated per microscopic field of view using ImageJ software.

### Isolation and culture of mouse primary macrophages and neutrophils

Six- to eight-week-old mice were euthanized via carbon dioxide inhalation. The tibias and femurs were harvested, longitudinally incised at both ends, and the bone marrow was gently flushed into pre-labeled centrifuge tubes with sterile PBS using 5-mL sterile syringes. The resulting cell suspension was centrifuged at 220 × g for 5 min at 4 °C. For the induction of bone marrow-derived primary macrophages (BMDMs), the cell pellets were resuspended in Dulbecco’s modified Eagle’s medium (DMEM, Gibco, USA) supplemented with 1% penicillin-streptomycin (Gibco, USA), 10% heat-inactivated fetal bovine serum (FBS, Gibco, USA), and 20 ng/mL macrophage colony-stimulating factor (M-CSF, PeproTech, USA). On the fourth day of culture, half the volume of the culture medium was replaced with fresh complete medium. On the seventh day, non-adherent cells and the supernatant were discarded; adherent BMDMs were then detached, counted, and seeded into culture plates at the desired density. Alternatively, bone marrow-derived primary neutrophils (BMDNs) were also isolated from the bone marrow cell suspension via density gradient centrifugation using commercial separation reagents (Solarbio, China). Isolated BMDNs were resuspended in RPMI 1640 medium (Hyclone, USA) containing 10% FBS, followed by cell counting and seeding. Given the relatively short lifespan of neutrophils, different stimulants were added to the culture 1 h later to allow for cell recovery, and the corresponding culture supernatants or cells were harvested 12 h post-stimulation. The drug administration protocols for the two cell types were as follows: DA (0.5 µM, 5 µM, 50 µM; Sigma-Aldrich, USA)(Alberio et al. 2011, Wang et al. 2019), SCH (10 µM; MCE, USA), etomoxir (ETO, 10 µM; MCE, USA), L‑carnitine (LCA, 5 mg/mL; MCE, USA), DNase I (5 µg/mL; Roche, Switzerland), AMG487 (2.5 µM), CXCL10 (100 ng/mL or 200 ng/ml), CXCL10 neutralizing antibody (αCXCL10) (4 µg/mL; R&D, USA), αIL-10 (400 ng/mL), recombinant mouse IL-10 (rIL-10, 100 ng/mL; PeproTech, USA) as well as their corresponding controls were administered 1 h prior to stimulation with LPS (100 ng/mL; InvivoGen, France) or IL-4 + IL-13 (20 ng/mL for both; Novoprotein, China). After a certain intervention period, cells and supernatants were collected for detection as needed.

### In vitro co-culture of macrophages and neutrophils

Two distinct macrophage-neutrophil co-culture protocols were utilized in this study. For conditioned medium-based indirect co-culture (Fig. 6C), BMDMs were seeded at an identical density (5 × 10⁵ cells/well) in equal volumes of culture medium and subjected to parallel treatment protocols for 24 h. Because all groups were processed under strictly matched conditions (same seeding density, same medium volume, and same culture duration), the resulting conditioned medium from each group directly reflects secretion per equivalent number of macrophages; therefore, further normalization was not performed. After treatment, the supernatants were collected and centrifuged (2000×g, 10 min, 4 °C) to remove cellular debris, and were then added to BMDNs (2.5 × 10⁶ cells/well) for 12 h of co-culture. For transwell-based direct cell co-culture (Fig. 6G), BMDMs (5 × 10⁵ cells/well) were first seeded in the upper inserts of 12-well transwell chambers (Corning, USA), followed by distinct treatments for 24 h. After thorough washing with PBS to remove residual drugs, 1.5 mL of medium was added to the upper chambers. Concurrently, BMDNs were seeded at a density of 2.5 × 10⁶ cells/well in the lower chambers containing 0.5 mL of medium. Co-cultures were then maintained for 12 h under standard cell culture conditions. Upon completion of the co-culture incubation, cell culture supernatants were collected. Adherent cells were harvested for subsequent analyses based on the experimental design.

### Human PBMC isolation and differentiation

Human PBMCs were isolated from 10 mL of venous blood samples collected from both ARDS patients and healthy donors. The samples were diluted with an equal volume of PBS and then carefully layered onto 3–5 mL of Ficoll separation medium (GE Healthcare, USA) to generate a clear, discrete gradient interface. Centrifugation was performed at 400×g for 20 min at room temperature with the centrifuge brake disengaged. Afterwards, the PBMC-enriched buffy coat fraction was gently harvested to avoid contamination with other cellular components. Isolated cells were washed with 5 volumes of PBS via centrifugation at 300×g for 10 min, then resuspended in RPMI-1640 medium supplemented with 10% heat-inactivated FBS. Cells were then plated in 12-well plates at a density of 1 × 10⁶ cells/mL and allowed to adhere for 24 h. Non-adherent cells were then removed by gentle washing, and the remaining adherent monocytes were cultured in fresh complete medium supplemented with 10 ng/mL human M-CSF (PeproTech, USA). Medium was refreshed on day 3, and cells were deemed mature macrophages after a 7-day culture period (Supplementary Fig. 9A). Differentiated macrophages were pretreated with DA (50 µM) for 1 h, followed by stimulation with LPS (100 ng/mL). Cells and supernatants were harvested 24 h after LPS exposure for downstream analysis.

### NETosis analysis

For NETosis analysis, the aforementioned BMDNs-seeded coverslips were subjected to immunofluorescence staining against Cit-H3 (Zenbio, R24572, 1:200, China) and MPO (Servicebio, GB11224; 1:500, China). Fluorescent imaging was performed on the Nikon Eclipse C1 fluorescence microscope (Nikon Corporation, Japan) equipped with a DS-U3 digital imaging system. For quantitative analysis of NETosis in in vitro systems, regions co-positive for MPO, Cit-H3, and DAPI were identified and quantified using ImageJ software. The proportion of NET-positive area was calculated as the ratio of MPO⁺Cit-H3⁺ area to the total DAPI-stained nuclear area(Tian et al. 2025). Identical fluorescence intensity thresholds were applied uniformly across all images and experimental groups to eliminate analytical bias. All image acquisition and quantitative analyses were performed in a blinded manner by investigators unaware of group allocations.

### Measurement of mitochondrial integrity and activity

BMDMs were plated in 4-well imaging chambers with cover-glass bottoms and subjected to the pre-designed experimental treatments for 24 h. Following treatments, cells were rinsed thrice with PBS to eliminate residual culture medium, then incubated with different fluorescent probes for targeted analyses. Specifically, MitoTracker Red CMXRos (Invitrogen, M7512, USA) was used for mitochondrial visualization, MitoSOX Green (Invitrogen, M36006, USA) for evaluating mitochondrial ROS levels, and DCFH-DA (Sigma-Aldrich, D6883, USA) for detecting total cellular ROS production. Cell nuclei were stained with DAPI (Beyotime, C1017, China) prior to confocal imaging. The images were acquired utilizing a Leica TCS SP8 system (Leica, Germany) and analyzed with ImageJ software. For each experimental sample, six non-overlapping areas were chosen at random. Within each area, quantification was performed for both the total fluorescence intensity and the number of cells present. Subsequently, the mean fluorescence intensity per cell was derived by normalizing total intensity to cell count, serving as an indicator of the target’s relative expression level.

### Transmission electron microscopic (TEM)

TEM was employed to examine mitochondrial morphology in macrophages exposed to various treatments. Cells were first fixed with 2.5% glutaraldehyde buffer at 4 °C for 24 h, then washed thoroughly with PBS. Subsequent steps included post-fixation in 1% osmium tetroxide for 1 h, dehydration using a gradient series of ethanol, and embedding in epoxy resin for curing. After preparing 70-nm ultrathin sections, the sections were stained with uranyl acetate and lead citrate before being imaged with a Hitachi HT7700 TEM (Tokyo, Japan). ImageJ software was used to perform form factor analysis for mitochondrial morphology assessment, with the form factor calculated as (perimeter²) / (4π × area).

### Public database processing

Transcriptomic data from a public pediatric ARDS cohort (GSE152978) was retrieved from the Gene Expression Omnibus database. After differential expression analysis to identify genes with significant expression changes, hierarchical clustering was conducted, and sample-wide expression patterns were visualized via heatmaps. Additionally, raw read counts were normalized to transcripts per million, and the expression levels of specific genes, including synthetic enzymes and receptors, were quantified and presented as bar charts.

### RNA sequencing (RNA-seq)

BMDMs from three independent biological replicates per condition (Control, LPS, LPS + DA) were seeded in 6‑cm dishes (5 × 10⁶ cells/dish) and treated for 12 h. Total RNA was extracted using TRIzol, and RNA integrity was assessed by an Agilent 2100 Bioanalyzer (RIN ≥ 8.0; Agilent Technologies, USA). Libraries were prepared using the TruSeq Stranded mRNA LT Sample Prep Kit (Illumina, USA) and sequenced on an Illumina NovaSeq 6000 platform (Macrogen, Korea) with 2 × 150 bp paired-end reads. Sequencing depth averaged raw reads per sample. Raw reads were filtered using fastp software to remove adapters and low-quality reads (Q < 20). High-quality clean reads were aligned to the mouse GRCm39 reference genome using HISAT2. Transcript assembly and quantification were performed with StringTie, and gene expression levels were calculated as FPKM values. Differential expression analysis between groups were conducted using edgeR. Genes with a fold change > 2 and Benjamini Hochberg adjusted p-value < 0.05 were considered significantly differentially expressed. For the heatmap in Fig. 4G, we selected the top 50 most variable genes across all samples (based on interquartile range of normalized counts) and performed hierarchical clustering using Euclidean distance and Ward’s linkage. Gene Ontology (GO) and Kyoto Encyclopedia of Genes and Genomes (KEGG) enrichment analyses were performed using clusterProfiler with an FDR cutoff of 0.05.

### Seahorse metabolic profiling assay

Seahorse extracellular flux (XF) assay was performed on the Seahorse XFe96 analyzer (Agilent Technologies, USA). BMDMs were seeded in Seahorse XFe96 assay plates and subjected to stimulation with DA (50 µM) and/or LPS (100 ng/mL) for 12 h. Prior to the assay, cells were rinsed and incubated for 1 h in glucose-, glutamine-, and pyruvate-free XF assay medium (pH 7.4) to standardize metabolic conditions. For the measurement of oxygen consumption rate (OCR), a key indicator of mitochondrial respiration, 2 µM oligomycin, 1 µM carbonyl cyanide 4-trifluoromethoxy phenylhydrazone (FCCP), and a mixture of 0.5 µM combination of rotenone and antimycin A were sequentially injected at predetermined time points to record real-time OCR dynamics. To specifically quantify OCR attributable to FAO, BMDMs were pretreated with palmitate-conjugated bovine serum albumin (palmitate-BSA) or vehicle control. The ratio of OCR values between palmitate-BSA-treated cells and untreated controls was calculated to evaluate the oxidation of exogenous palmitate, thereby determining cellular FAO activity.

### Statistical analysis

Data analyses were conducted with GraphPad Prism 9.5.1 (GraphPad Software, Inc., San Diego, USA). The Shapiro-Wilk test was used to verify data normality. Normally distributed data were analyzed via two-tailed unpaired Student’s t-test (two groups) or one-way ANOVA with Bonferroni post-hoc test (multiple groups). Whereas non-normal continuous variables were analyzed by rank-sum test, and chi-square test was applied for categorical variables among different patient treatment groups. In addition, Pearson’s correlation test was employed to evaluate the correlation among variables. All data presented in this study are derived from biological replicates, defined as independent animals or independently cultured cell passages. Experiments were independently replicated at least three times. All independent experiments yielded similar results, and data were pooled only after confirming no significant batch‑to‑batch variation. Statistical analyses were performed based on the number of biological replicates (n). Results are presented as mean ± SEM and median (interquartile range) accordingly, with *p* < 0.05 considered statistically significant. Detailed statistical procedures are provided in the corresponding figure legends.

Cell viability detection, immunofluorescence assay, immunoblotting detection, enzyme-linked immunosorbent assay (ELISA), RNA extraction and reverse transcription, and quantitative polymerase chain reaction (qPCR) were performed as described in detail in the Supplementary Materials-Methods.

## Results

### Analysis of the dopaminergic dynamic variability in ALI models

**Fig. 1:**
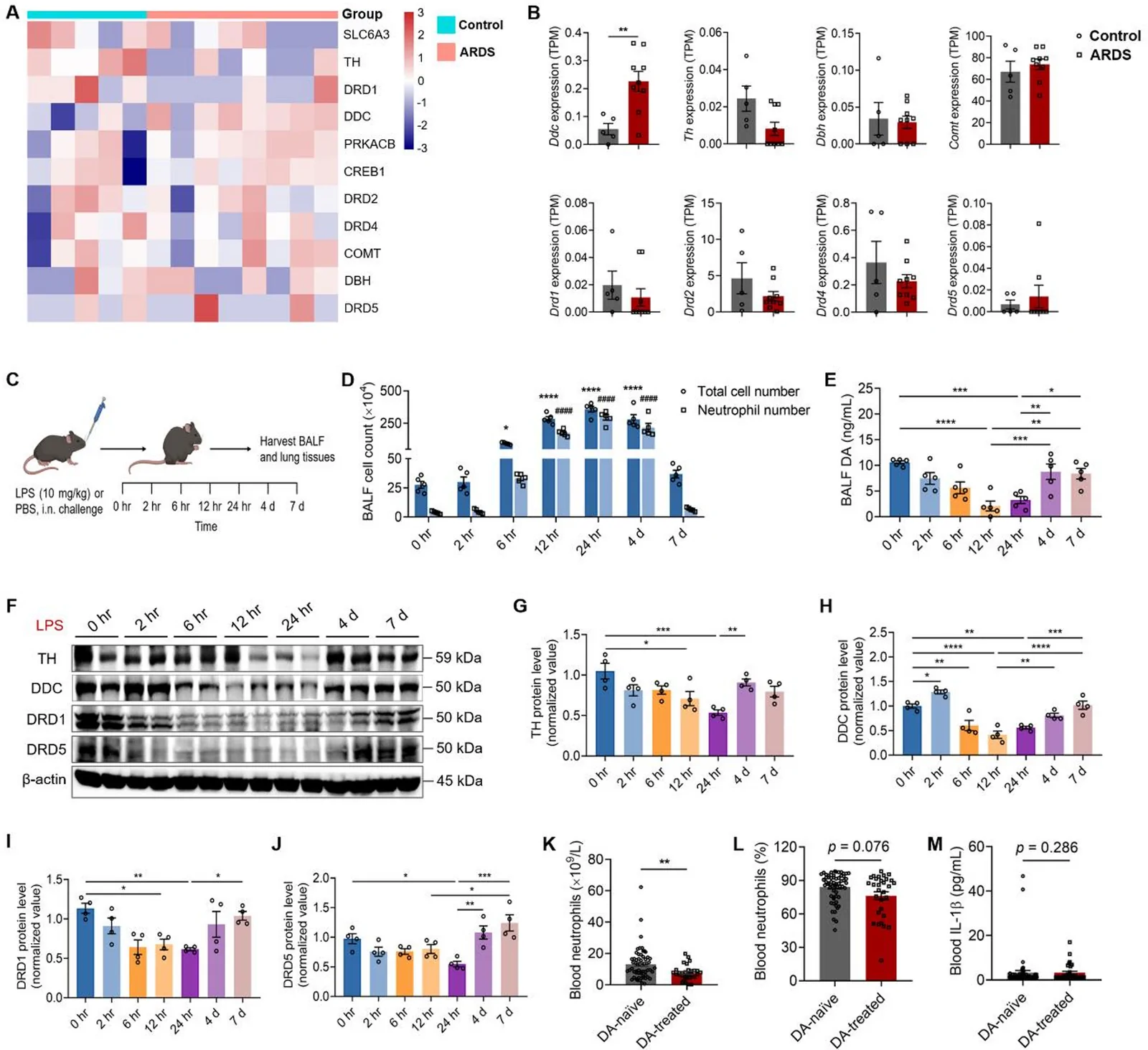
Dynamic changes of dopaminergic signaling during the course of ALI. **A** Hierarchical clustering heatmap of core dopaminergic gene expression in the public pediatric acute respiratory distress syndrome (ARDS) cohort (GSE152978, human nasal brushings), showing distinct signatures between the control (*n* = 5) and ARDS (*n* = 8) groups. **B** Quantitative analysis of dopaminergic gene expression in (**A**) (presented as transcripts per million, TPM). **C** Experimental schematic: mice were intranasally administered lipopolysaccharide (LPS,10 mg/kg) or phosphate-buffered saline (PBS), with time-series sampling of bronchoalveolar lavage fluid (BALF) or lung tissues at 0–7 days. **D**,** E** Total cell count, neutrophil number (**D**), and level of dopamine (DA) in mouse BALF were analyzed at different time points after LPS stimulation (*n* = 5 per group). **F-J** The protein levels of key enzymes for DA synthesis tyrosine hydroxylase (TH), DA decarboxylase (DDC), and D1-like receptors DRD1, DRD5 were determined (**F**) and quantified (**G**-**J**) in mouse lung tissues (*n* = 4 per group). Band intensities were normalized to β-actin. **K-M** Hematologic parameters in DA-naïve vs. DA-treated ARDS patients, including neutrophil count (**K**), neutrophil percentage (**L**), and IL-1β secretion (**M**) (*n* = 60 for DA-naïve while *n* = 30 for DA-treated group). Data are presented as mean ± SEM and analyzed with a 95% confidence interval. Statistical analysis was performed using Mann-Whitney U-test (**K**-**M**), two-tailed unpaired Student t test or one-way ANOVA followed by Bonferroni’s post hoc test. *p < 0.05, **p < 0.01, ***p < 0.001, ****p < 0.0001; ####*p* < 0.0001 for neutrophil number, compared with the related control (0 h). “n” represents the number of independent biological replicates

To characterize the dopaminergic system dynamics within a clinically relevant framework, we first integrated nasal brushings transcriptomic data from a public pediatric ARDS cohort (GSE152978, nasal brushings) to examine the expression of core dopaminergic genes. Hierarchical clustering revealed a distinct reprogramming signature in human ARDS samples (Fig. 1A, B): *Ddc* (dopa decarboxylase) was significantly upregulated, while its upstream counterpart *Th* (tyrosine hydroxylase) showed a downward trend. Concomitantly, DA receptor expression tended to be remodeled, whereas key metabolic genes (*Dbh*, *Comt*) remained unchanged. This parallel transcriptional shift between human ARDS and murine ALI models points toward an adaptive recalibration of dopaminergic signaling during ALI, providing a critical clinical correlate for our subsequent mechanistic investigations in the murine model.

To characterize the dopaminergic system dynamics during LPS-induced ALI, we established a murine model with intranasal instillation of LPS (10 mg/kg) and performed time-series sampling of BALF and lung tissues (Fig. 1C). Analysis of BALF cellularity revealed a rapid influx of total cells and neutrophils post-LPS, peaking at 24 h before a gradual decline (Fig. 1D). Intriguingly, DA levels in BALF exhibited a striking inverse temporal relationship with neutrophil infiltration (Fig. 1E). DA concentrations dropped significantly during the early phase (12–24 h), reaching a nadir concurrent with peak neutrophil counts, and subsequently recovered at days 4 and 7. Through correlation analysis, we further demonstrated that DA levels are negatively correlated with airway inflammatory cells, particularly neutrophils (Supplementary Fig. 1A, B). This coordinated response was also supported by immunoblot analysis of key dopaminergic proteins in lung tissue (Fig. 1F). The expression levels of DA receptors DRD1 and DRD5, along with the key biosynthetic enzyme tyrosine hydroxylase (TH) and DA decarboxylase (DDC), showed dynamic changes that paralleled the fluctuations in BALF DA and neutrophil counts. Quantitative analysis confirmed significant time-dependent alterations in TH, DDC, DRD1, and DRD5 protein levels (Fig. 1G-J).

To assess clinical relevance, we conducted a retrospective, observational, non-randomized analysis comparing hematologic parameters between ARDS patients with or without DA treatment (Supplementary Table 2). While absolute neutrophil counts differed significantly between groups (Fig. 1K), the percentage of neutrophils, and level of proinflammatory cytokine showed a decreasing trend (Fig. 1L, M). Of note, these clinical data are observational in nature, and DA administration in this setting was not randomized. Collectively, these parallel findings from murine models and patient data suggest that dynamic regulation of the dopaminergic system is integral to neutrophil-mediated pulmonary inflammation, revealing a multi-layered regulatory axis in ALI/ARDS.

### DA treatment attenuates neutrophil-driven inflammation in the ALI mouse model via macrophage-dependent mechanism

**Fig. 2:**
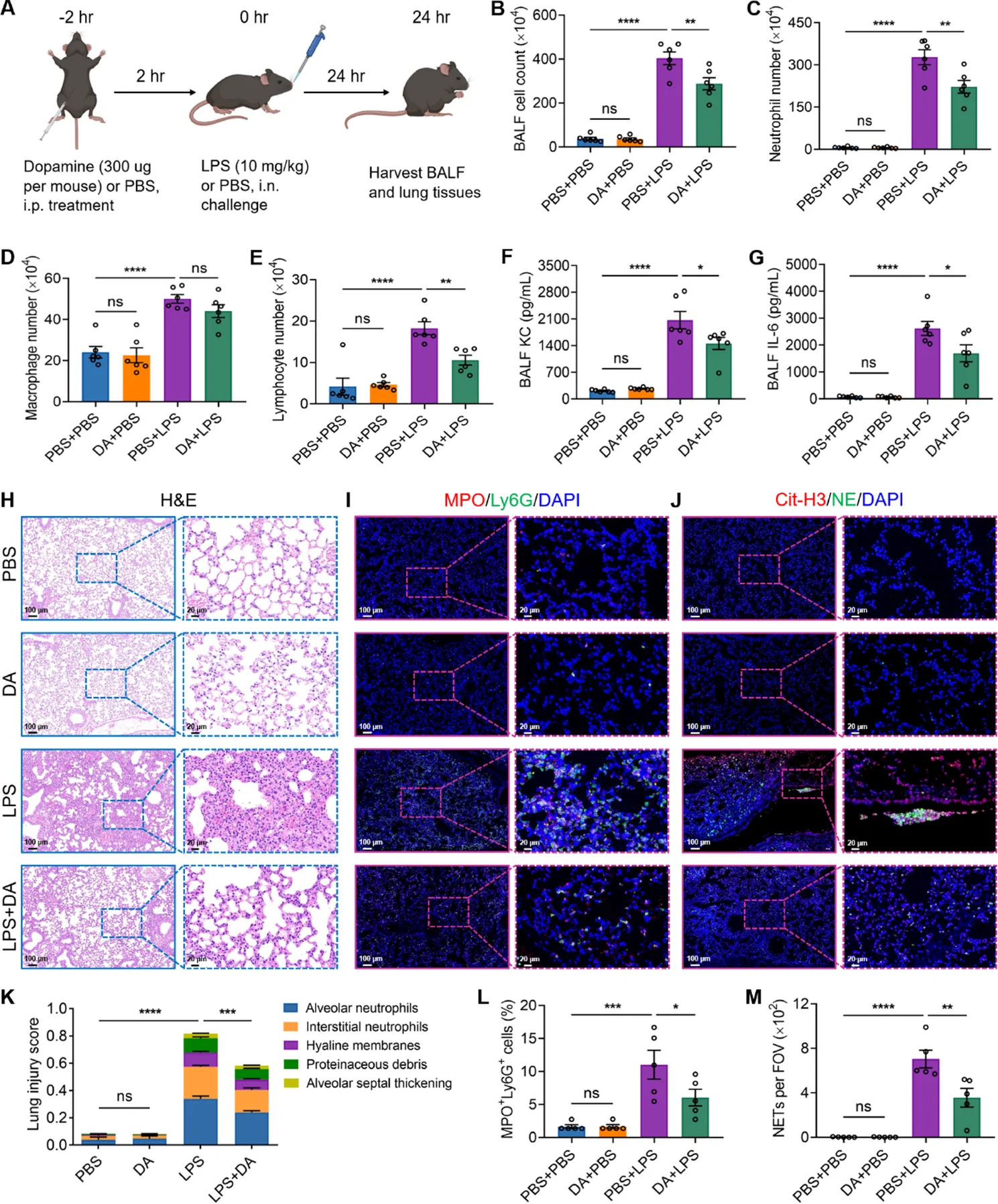
DA administration attenuates neutrophilic inflammation in the ALI mouse model. **A** Experimental schematic: mice were pretreated with an intraperitoneal injection of DA (300 µg per mouse) or equal amount of PBS 2 h prior to an intranasal challenge with LPS (10 mg/kg) or PBS. Samples were harvested 24 h post-LPS challenge. **B-E** Quantification of BALF cellular components in experimental groups, including total cell count (**B**), neutrophils (**C**), macrophages (**D**), and lymphocytes (**E**) (*n* = 6 per group). **F**,** G** Levels of chemokine KC (F) and cytokine IL-6 (G) in BALF from distinct groups (*n* = 6 per group). **H**,** K** Representative hematoxylin and eosin (H&E)-stained lung tissue sections (**H**) and corresponding injury scoring (**K**) at 24 h post-LPS challenge (*n* = 5 per group). Scale bar: 100 μm in the left panel, 20 μm in the right panel. **I**,** L** Representative immunofluorescence staining of lung tissues for myeloperoxidase (MPO, shown in red) and Ly6G (shown in green) (**I**), with quantification of double-positive cells shown in (**L**) (*n* = 5 per group). Nuclei were stained with DAPI, displayed in blue. Scale bar: 100 μm in the left panel, 20 μm in the right panel. **J**,** M** Representative immunofluorescence images showing neutrophil extracellular traps (NETs) in lung tissues, identified by colocalization of citrullinated histone H3 (Cit‑H3) and neutrophil elastase (NE) (Cit‑H3⁺/NE⁺) (J; Cit-H3 shown in red, NE shown in green), along with the corresponding NETs quantification (**M**) (*n* = 5 per group). Nuclei were stained with DAPI, displayed in blue. Scale bar: 100 μm in the left panel, 20 μm in the right panel. All immunofluorescence staining quantification was performed in at least 6 random fields per section. Data are presented as mean ± SEM and analyzed with a 95% confidence interval. Statistical analysis was performed using one-way ANOVA followed by Bonferroni’s post hoc test. *p < 0.05, **p < 0.01, ***p < 0.001, ****p < 0.0001, ns, not significant. “n” represents the number of independent biological replicates

To evaluate the impact of DA on LPS-induced ALI model, mice were pretreated with DA prior to intranasal LPS challenge (Fig. 2A). Analysis of BALF at 24 h revealed that DA administration significantly reduced the LPS-induced increase in total cell counts, especially neutrophil and lymphocyte numbers (Fig. 2B-E). Consistent with its effect on cellular infiltration, DA pretreatment significantly suppressed the elevated chemokine, KC and pro-inflammatory cytokine, IL-6, in BALF of ALI mice (Fig. 2F, G). DA did not alter baseline lung histology in PBS-treated animals. In contrast, following LPS challenge, DA pretreatment significantly mitigated the progressive tissue damage observed 24 h after LPS induction (Fig. 2H, K). To further investigate the effect of DA on neutrophil activation, we performed immunostaining for specific markers for neutrophil presence (Ly6G), granule enzymes (MPO, NE), and NETosis (Cit-H3). As shown in Fig. 2I, J, L, M, LPS challenge robustly increased the expression of MPO, NE and Cit-H3 in lung-infiltrating neutrophils, while DA treatment attenuated the expression of these markers, indicating a suppression of neutrophil activation and associated inflammatory cascades. In summary, these findings demonstrate that DA contribute to the attenuation of LPS-induced ALI by limiting neutrophil infiltration and suppressing their pro-inflammatory activation state. To further determine the role of neutrophils in DA-mediated protective effects in ALI mice, we specifically depleted neutrophils using a neutralizing antibody (αLy6G), with depletion efficacy verified by immunofluorescence for lung tissue (Supplementary Fig. 2A, B). The inhibitory ability of DA in LPS-induced inflammation was observed in isotype control-treated mice but absent in neutrophil-depleted group, evidenced by airway total cell number, neutrophil count, BALF KC level, and lung injury degree (Supplementary Fig. 2C-G).

**Fig. 3:**
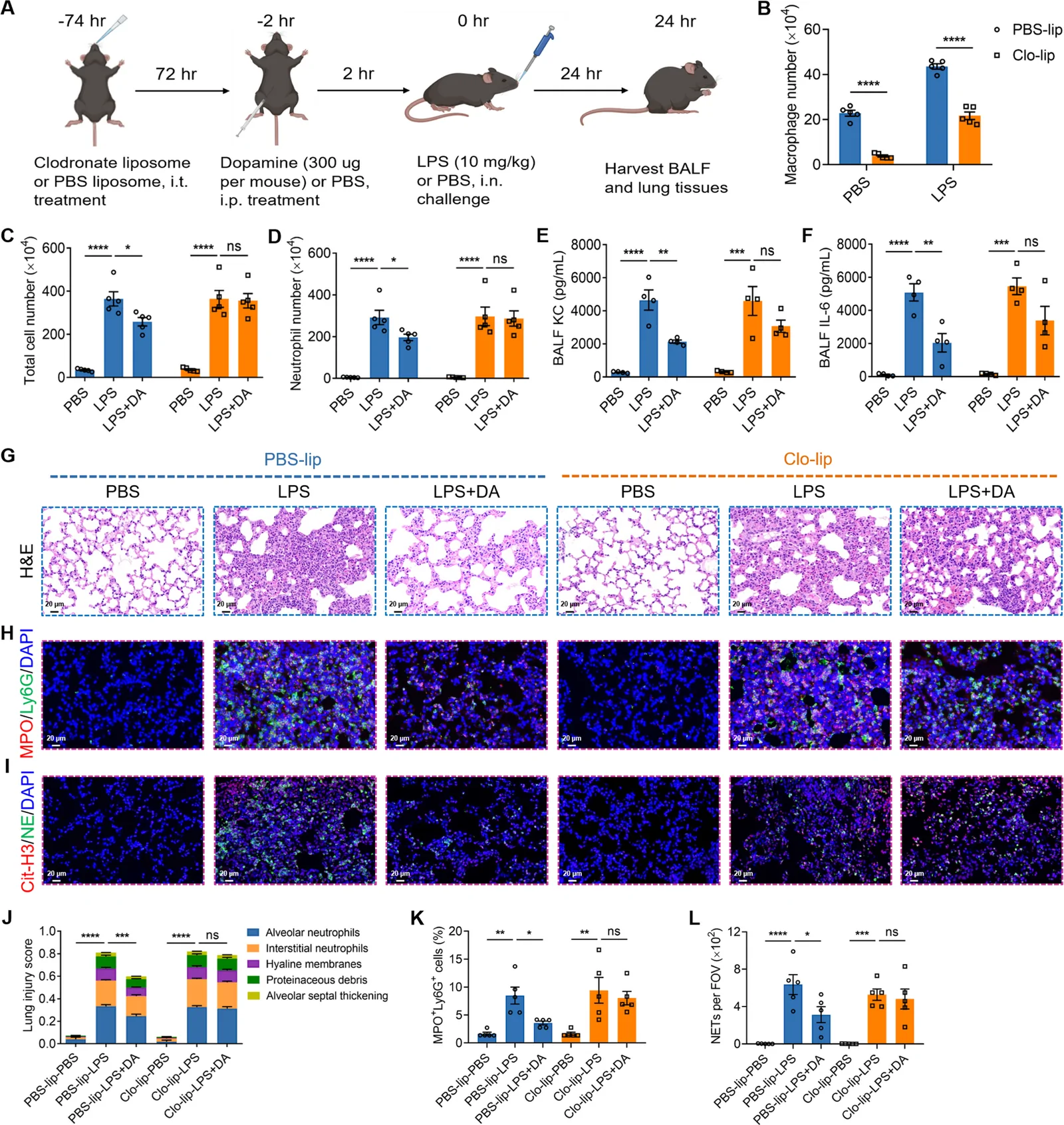
DA-mediated protection against ALI requires macrophage-dependent mechanisms. **A** Experimental schematic: to deplete lung macrophages, mice received an intratracheal administration of clodronate liposome (Clo-lip) or PBS-lip (5 mg/mL, 75 µL per mouse) 74 h prior to LPS challenge. Mice were then treated with an intraperitoneal injection of DA (300 µg per mouse) or PBS 2 h before the intranasal instillation of LPS (10 mg/kg) or PBS. All samples were harvested 24 h post-LPS challenge. **B-D** Quantification of BALF cellular components in experimental groups, including macrophages (**B**), total cell count (**C**), and neutrophils (**D**) (*n* = 5 per group). **E**,** F** Levels of chemokine KC (**E**) and cytokine IL-6 (**F**) in BALF from distinct groups (*n* = 4 per group). **G**,** J** Representative H&E-stained lung tissue sections (**G**) and corresponding injury scoring (**J**) at 24 h post-LPS challenge (*n* = 5 per group). Scale bar: 20 μm. **H**,** K** Representative immunofluorescence staining of lung tissues for MPO (shown in red) and Ly6G (shown in green) (**H**), with quantification of double-positive cells shown in (**K**)). Nuclei were stained with DAPI, displayed in blue. Scale bar: 20 μm. **I**,** L** Representative immunofluorescence images showing Cit-H3⁺/NE⁺ NETs in lung tissues (I; Cit-H3 shown in red, NE shown in green), along with the corresponding NETs quantification (**L**) (*n* = 5 per group). Nuclei were stained with DAPI, displayed in blue. Scale bar: 20 μm. All immunofluorescence staining quantification was performed in at least 6 random fields per section. Data are presented as mean ± SEM and analyzed with a 95% confidence interval. Statistical analysis was performed using two-tailed unpaired Student t test or one-way ANOVA followed by Bonferroni’s post hoc test. *p < 0.05, **p < 0.01, ***p < 0.001, ****p < 0.0001, ns, not significant. “n” represents the number of independent biological replicates

Having observed that DA suppresses neutrophil-driven inflammation, we next sought to determine whether this protective effect is mediated through the upstream macrophages. To address this, we depleted macrophages using Clo-lip (Fig. 3A). Successful depletion was confirmed by BALF cell counting (Fig. 3B). In PBS-lip control mice, DA pretreatment significantly attenuated LPS-increased numbers of BALF total cells and neutrophils. However, this effect was completely abrogated in macrophage-depleted (Clo-lip) mice (Fig. 3C, D). Similarly, DA-mediated reductions in the pro-inflammatory cytokines KC and IL-6 were lost upon macrophage depletion (Fig. 3E, F). Histopathological analysis and assessment of neutrophil activation and NETosis consistently showed that DA alleviated lung injury and suppressed neutrophilic inflammation in a macrophage-dependent manner (Fig. 3G-L). Taken together, these loss-of-function experiments demonstrate that both macrophages and neutrophils are essential cellular mediators required for DA to protect against LPS-induced ALI, with the underlying mechanism yet to be elucidated.

### DA inhibits pro-inflammatory signaling in macrophages via the D1-like receptors

**Fig. 4:**
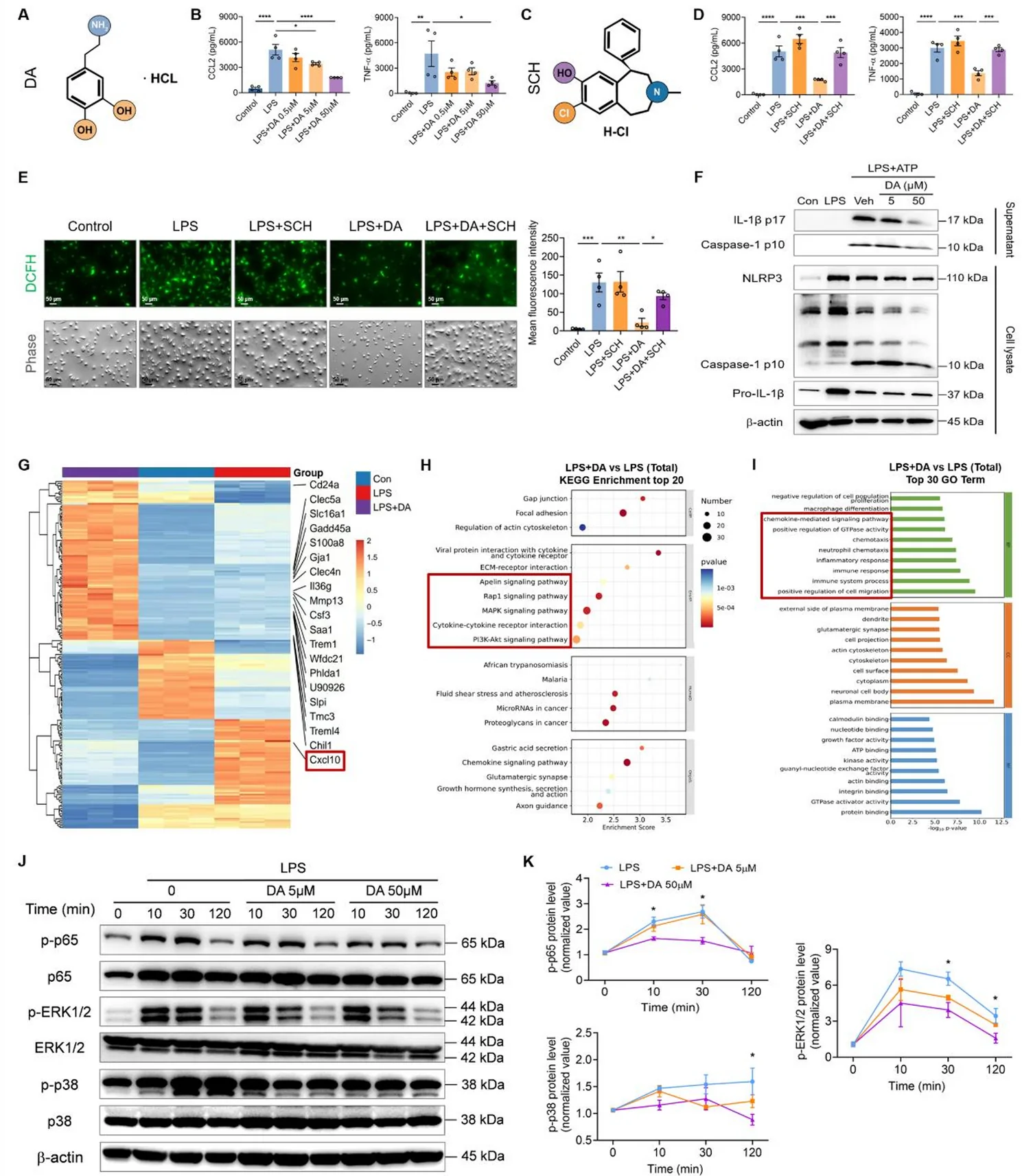
DA suppresses inflammasome activation and pro-inflammatory signaling in BMDMs via D1-like receptors. **A** Chemical structure of DA. **B** Secretion of pro-inflammatory cytokines CCL2 and TNF-α in bone marrow-derived macrophages (BMDMs) treated with different concentrations of DA and/or LPS (*n* = 4 per group). **C** Chemical structure of SCH23390 (SCH). **D **Secretion of pro-inflammatory cytokines CCL2 and TNF-α in BMDMs treated with DA, SCH, and/or LPS (*n* = 4 per group). **E** Representative images of intracellular reactive oxygen species (ROS) staining (DCFH-DA probe, shown in green) with corresponding brightfield views, and analysis quantified as the mean fluorescence intensity across treatment groups (*n* = 4 per group). Scale bar: 50 μm. **F** Immunoblotting detection of key endpoints of NLRP3 inflammasome activation, including NLRP3 expression, Caspase-1 activation, and IL-1β maturation and secretion in the presence of DA at indicated concentrations (*n* = 3 per group). **G** A heatmap of the most different expressed genes in BMDMs among distinct groups. **H**,** I** KEGG (**H**) and GO (**I**) enrichment analyses were conducted on the different expressed genes between LPS + DA versus LPS group (*n* = 3 per group). **J**,** K** Immunoblotting (**J**) with quantitative analysis (**K**) was used to assess the regulatory effects of DA on the phosphorylation of key proteins: p65 in the NF-κB pathway, and ERK1/2, p38 in the MAPK pathway (*n* = 3 per group). Band intensities were normalized to β-actin. All immunofluorescence staining quantification was performed in at least 6 random fields per section. Data are presented as mean ± SEM and analyzed with a 95% confidence interval. Statistical analysis was performed using two-tailed unpaired Student t test or one-way ANOVA followed by Bonferroni’s post hoc test. *p < 0.05, **p < 0.01, ***p < 0.001, ****p < 0.0001. *p < 0.05, LPS + DA 50 µM versus LPS group (**K**). “n” represents the number of independent biological replicates

Building on the pivotal roles of macrophages and neutrophils as targets of DA, we next assessed its immunomodulatory effects on these primary cells. We found that DA dose-dependently suppressed LPS-induced production of pro-inflammatory cytokines CCL2, TNF-α in BMDMs without cytotoxicity at tested concentrations (0.5–50 µM), but had no direct effect on BMDNs (Fig. 4A, B; Supplementary Fig. 3A-D). We therefore focused subsequent investigation on macrophage regulation. Based on our previous finding that the D1-like receptor agonist SKF38393 alleviates inflammation in THP-1 macrophages(Meng et al. 2023), we hypothesized that DA exerts its anti-inflammatory effects through the same receptor class. To test this, we employed the specific D1 receptor antagonist SCH (Fig. 4C). Crucially, the inhibition of DA on cytokine secretion was completely abolished by co-treatment with SCH (10 µM; Fig. 4D; Supplementary Fig. 3E), confirming the essential role of D1-like receptor signaling. Furthermore, DA attenuated LPS-induced ROS overproduction, effects that were also reversed by SCH (Fig. 4E). These results demonstrate that DA suppresses LPS-induced inflammation and oxidative stress specifically via the D1-like receptors. To further explore the molecular mechanisms underlying DA-mediated regulation of inflammatory responses, we first examined the effect of DA on inflammasome assembly in LPS-primed cells. As shown in Fig. 4F, co-treatment with DA (50 µM) significantly inhibited LPS-triggered NLRP3 inflammasome activation, Caspase-1 cleavage, and release of IL-1β.

To systematically identify DA-modulated transcripts, we performed RNA-seq analysis on BMDMs from different groups. The heatmap revealed distinct gene expression patterns among groups: LPS stimulation altered the expression of multiple inflammation-related genes, while DA treatment reversed the dysregulation of key genes, such as *Cxcl10* (Fig. 4G). KEGG enrichment analysis between LPS + DA and LPS groups indicated that DA primarily regulated pathways involved in MAPK signaling cascade and cytokine-cytokine receptor interaction (Fig. 4H). GO enrichment analysis further confirmed that DA targeted genes associated with chemokine-mediated signaling pathway, neutrophil chemotaxis, and inflammatory processes (Fig. 4I), collectively illustrating DA’s coordinated inhibition of pro-inflammatory signaling networks. We next validated the effect of DA on MAPK/NF-κB signaling, which is critical for inflammatory activation (Fig. 4J, K). LPS stimulation rapidly induced phosphorylation of p65 (NF-κB), ERK1/2, and p38 within 30 min, which could be significantly attenuated by DA (50 µM) in a time-dependent manner. In summary, our findings validate the hypothesis that DA engages macrophage D1-like receptors to attenuate LPS-induced inflammation, primarily by inhibiting the MAPK/NF-κB signaling axis, which in turn suppresses downstream pro-inflammatory gene expression, NLRP3 inflammasome activation, and oxidative stress.

### DA promotes anti-inflammatory macrophage polarization through maintaining mitochondrial FAO and homeostasis

**Fig. 5:**
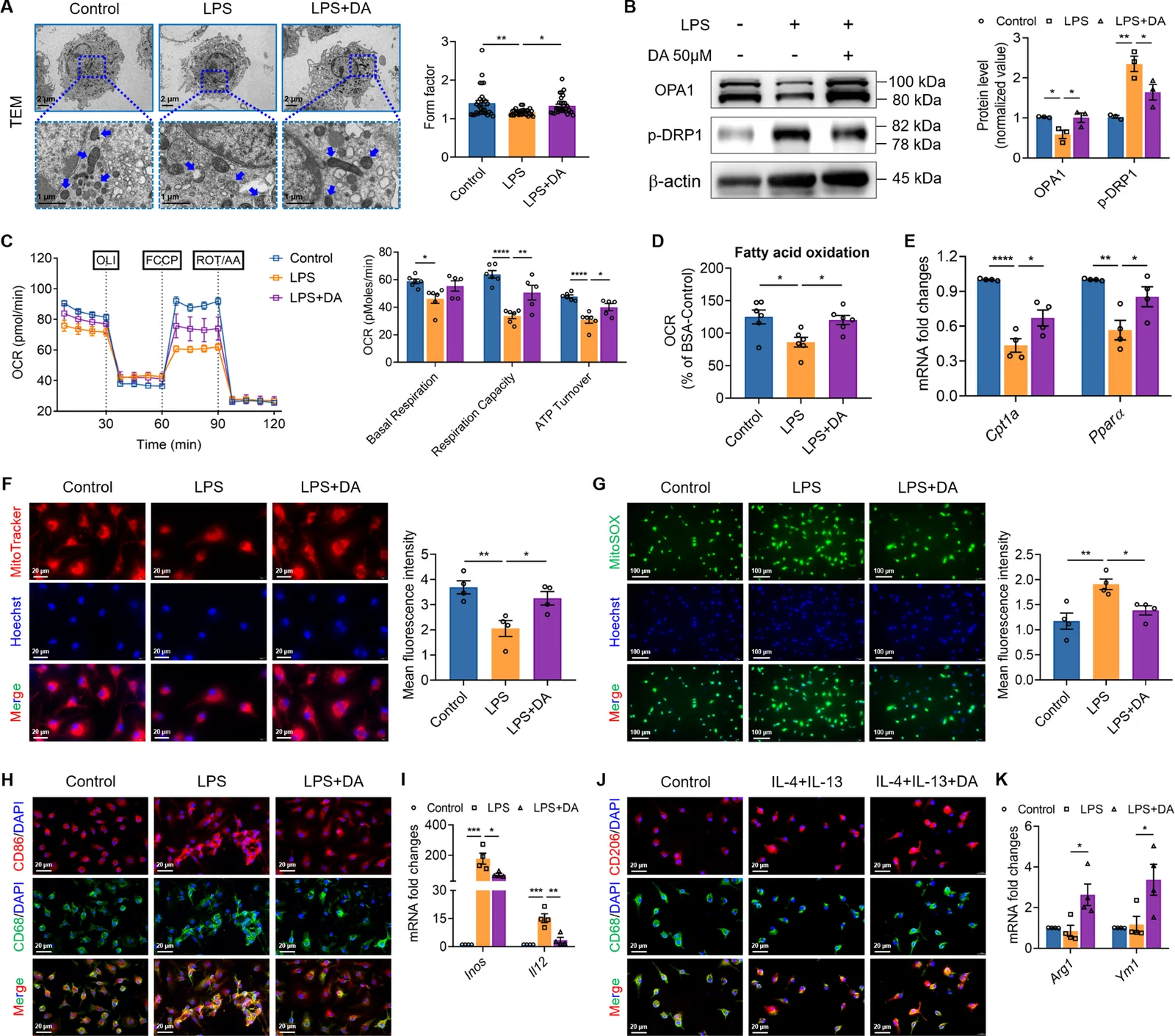
DA promotes anti-inflammatory M2 polarization through enhancing mitochondrial FAO and homeostasis. **A** Representative TEM images displayed mitochondrial morphology in BMDMs under different treatments, with mitochondria indicated by blue arrowheads. Scale bars: 2 μm for upper panel, 1 μm for lower panel. The form factor (Perimeter² / [4π × Area]), a parameter of mitochondrial morphology, was quantified (*n* = 30 per group from 4 independent biological experiments). **B** Immunoblot assay exhibiting the expressions of fusion protein OPA1 and fission protein p-DRP1 in BMDMs under different treatments (*n* = 3 per group). Band intensities were normalized to β-actin. **C**,** D** Oxygen consumption rate (OCR), indicated by basal respiration (*n* = 6, 6, 5), respiration capacity (*n* = 6, 6, 5), and ATP turnover (*n* = 6, 6, 5) (**C**), as well as fatty acid oxidation (FAO) level (D, *n* = 6 per group) of BMDMs from different treatment groups were measured on a Seahorse XFe96 analyser. **E** Induction of *Cpt1a* and *Pparα* mRNA expression in BMDMs was estimated by qPCR detection (*n* = 4 per group). **F**,** G** Representative images and quantitative analysis of BMDMs stained with MitoTracker (F, shown in red) and MitoSOX (G, shown in green) probes upon different treatments (*n* = 4 per group). The nuclei were counterstained with Hoechst (shown in blue). Scale bars: 20 μm for MitoTracker probe, 100 μm for MitoSOX probe. **H**,** J** Immunofluorescence staining for M1 (CD68/CD86, H) and M2 (CD68/CD206, J) markers in BMDMs under various treatments (*n* = 4 per group). Nuclei were stained with DAPI, displayed in blue. Scale bar: 20 μm. Quantification was performed in at least 6 random fields per group. **I**,** K** Induction of expressions of M1 markers *Inos*, *Il12* (**I**), and M2 markers *Arg1*, *Ym1* (**K**) in BMDMs after indicated treatments was determined via qPCR assay (*n* = 4 per group). All gene expression levels were normalized to *β-actin*. Data are presented as mean ± SEM and analyzed with a 95% confidence interval. Statistical analysis was performed using one-way ANOVA followed by Bonferroni’s post hoc test. *p < 0.05, **p < 0.01, ***p < 0.001, ****p < 0.0001. “n” represents the number of independent biological replicates

Considering that cellular metabolic reprogramming is a key determinant of macrophage phenotype(Wang et al. 2023, Kelly and O’Neill 2015), we next examined whether DA’s anti-inflammatory signaling translates into improved mitochondrial homeostasis and a shift in polarization state. First, TEM revealed that DA treatment restored the mitochondrial architecture, including swelling and cristae disorganization caused by LPS challenge (Fig. 5A). This structural repair was accompanied by a rebalancing of mitochondrial dynamics(Harrington et al. 2023, Archer 2013), as DA normalized the LPS-induced imbalance in fusion- and fission-related proteins, OPA1 and p-DRP1 (Fig. 5B). As the energy metabolism factory of the cell, we next assessed mitochondrial metabolic function. LPS stimulation suppressed both general mitochondrial respiration and FAO-linked OCR. DA treatment not only restored these parameters (Fig. 5C, D), but also reversed the LPS-induced downregulation of key FAO regulators, *Cpt1a* and *Pparα*, at the mRNA level (Fig. 5E). Moreover, DA also mitigated LPS-triggered mitochondrial network fragmentation and scavenged mitochondrial ROS (Fig. 5F, G), collectively demonstrating that DA enhances mitochondrial FAO and redox homeostasis.

Given the established role of FAO in driving anti-inflammatory M2 macrophage polarization and maintaining mitochondrial homeostasis(Nomura et al. 2016, Vats et al. 2006), we hypothesized that DA-mediated FAO enhancement would shift macrophage phenotype. In LPS-stimulated BMDMs, DA reduced the expression of the M1 surface marker CD86 and downregulated the mRNA levels of *Inos* and *Il12* (Fig. 5H, I). Conversely, in an IL-4/IL-13-induced M2 model, DA increased the expression of the M2 marker CD206 and further elevated the mRNA levels of *Arg1* and *Ym1* (Fig. 5J, K). In summary, DA repairs mitochondrial structure and function, enhances FAO, and consequently skews macrophages toward an anti-inflammatory M2 phenotype, thereby resolving inflammation.

### DA regulates neutrophil inflammatory response and NETosis via macrophage-mediated crosstalk

**Fig. 6:**
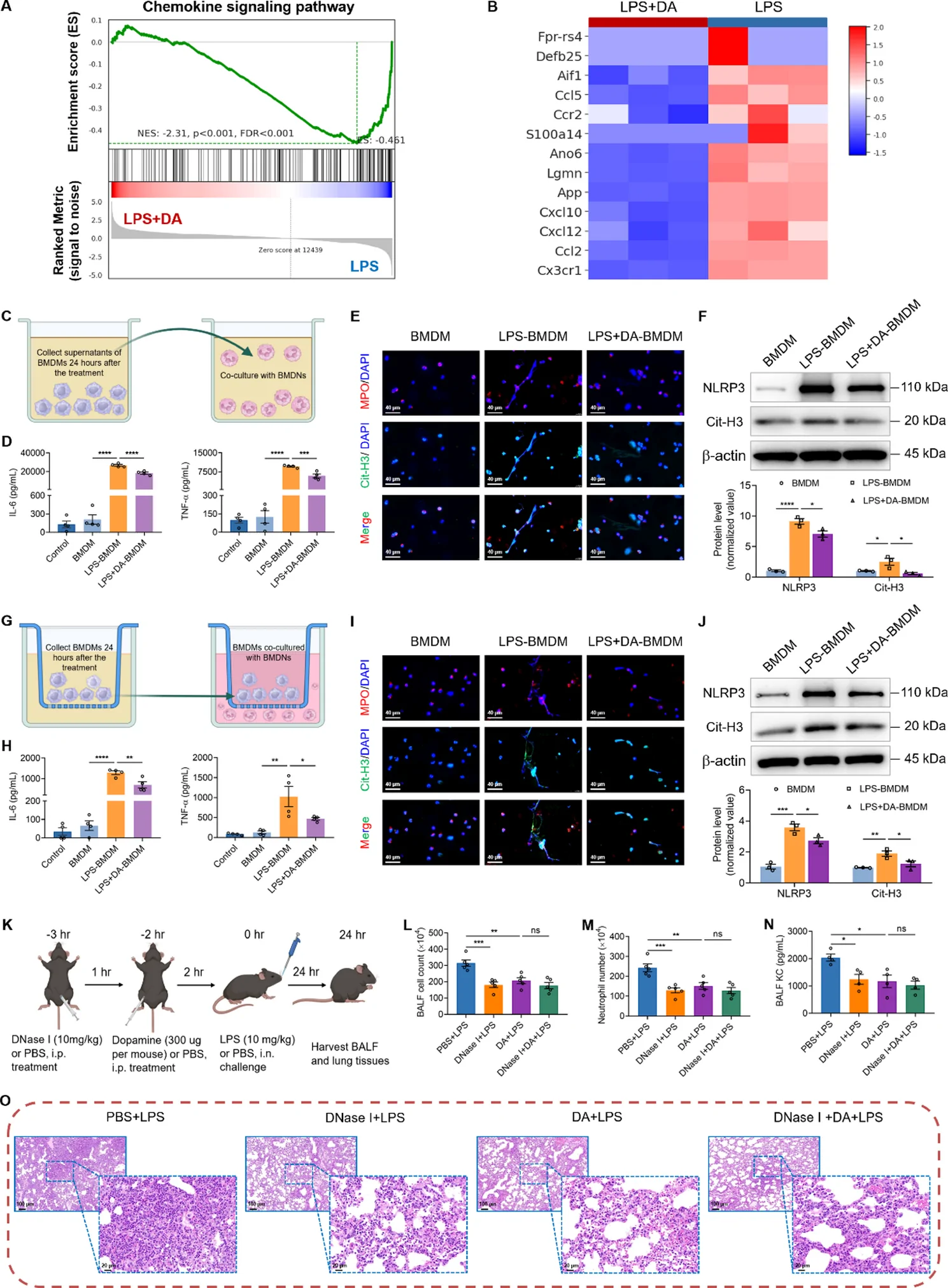
DA curbs neutrophil inflammatory response and NETosis through macrophage-neutrophil crosstalk in ALI. **A**,** B** Gene set enrichment analysis (**A**) and the representative heatmap (**B**) showing the downregulated genes related to chemokine signaling pathway between the two groups. **C** Schematic of the conditioned medium co-culture system. Supernatants were collected from BMDMs under control, LPS, or LPS + DA conditions and then incubated with neutrophils for 12 h. **D** Levels of IL-6 and TNF-α in supernatants from the indirect co-culture system (*n* = 4 per group). **E** Representative immunofluorescence images of NETosis in bone marrow-derived neutrophils (BMDNs). Cells from the indirect co-culture system were stained for the NETs markers MPO (shown in red) and Cit-H3 (shown in green) (*n* = 4 per group). Nuclei were stained with DAPI, displayed in blue. Scale bar: 40 μm. **F** Immunoblot exhibiting the expressions of NLRP3 and Cit-H3 in BMDNs from the indirect co-culture system (*n* = 3 per group). **G** Schematic of the transwell-based direct co-culture system. BMDMs and BMDNs were co-cultured using a transwell system for 12 h, with BMDMs seeded in the upper chamber and BMDNs in the lower chamber. **H** Levels of IL-6 and TNF-α in supernatants from the direct co-culture system (*n* = 4 per group). **I** Representative immunofluorescence images of NETosis in BMDNs. Cells from the direct co-culture system were stained for the MPO (shown in red) and Cit-H3 (shown in green) (*n* = 4 per group). Nuclei were stained with DAPI, displayed in blue. Scale bar: 40 μm. **J** Immunoblot exhibiting the expressions of NLRP3 and Cit-H3 in BMDNs from the direct co-culture system (*n* = 3 per group). **K** Experimental schematic: mice were pretreated with an intraperitoneal injection of DNase I (10 mg/kg) or PBS 3 h prior to LPS challenge, followed by DA (300 µg per mouse) or PBS 1 h later. All samples were harvested 24 h post-LPS (10 mg/kg) instillation. **L-N** Total cell count (L, *n* = 5 per group), neutrophil number (M, *n* = 5 per group), and KC level (N, *n* = 4 per group) in BALF were quantified across groups. **O** Representative H&E-stained lung sections from different experimental groups (*n* = 5 per group). Scale bar: 100 μm in the upper panel, 20 μm in the lower panel. Band intensities were normalized to β-actin. Quantification of NET-positive areas was performed in at least 6 random fields per group. Data are presented as mean ± SEM and analyzed with a 95% confidence interval. Statistical analysis was performed using one-way ANOVA followed by Bonferroni’s post hoc test. *p < 0.05, **p < 0.01, ***p < 0.001, ****p < 0.0001, ns, not significant. “n” represents the number of independent biological replicates

While DA showed limited direct effects on neutrophils, which play an effector role in ALI, we hypothesized that it might regulate their activity indirectly via macrophages. We further analyzed the sequencing data of the aforementioned BMDMs. Gene set enrichment analysis identified a downregulation in the chemokine signaling pathway and associated genes (Fig. 6A, B). To address this, we next investigated whether DA modulates macrophage-neutrophil crosstalk using in vitro co-culture systems (Fig. 6C, G). In an indirect co‑culture system containing a fixed number of neutrophils (thereby excluding neutrophil recruitment), conditioned medium from LPS‑stimulated BMDMs markedly activated BMDNs. This activation elevated their secretion of IL‑6 and TNF‑α (Fig. 6D) and induced NETosis, a process that critically depends on Cit-H3‑mediated chromatin decondensation. Notably, pretreating the macrophages with DA significantly attenuated all these effects (Fig. 6E; Supplementary Fig. 4A, B). Mechanistically, conditioned medium from DA‑treated BMDMs suppressed the expression of NLRP3 and Cit-H3 in neutrophils, indicating that DA exerts its macrophage‑mediated inhibitory effect on LPS‑induced neutrophil over‑activation at least in part through downregulating these key molecules (Fig. 6F). Meanwhile, a direct transwell co‑culture yielded consistent results (Fig. 6H-J; Supplementary Fig. 4C, D). In contrast, DA exerted no such effect when directly administered to neutrophils, indicating its action is primarily mediated through macrophages (Supplementary Fig. 4E, F).

To translate these in vitro findings into a physiological context, we established an LPS-induced ALI model in mice pretreated with DA, DNase I (to enzymatically degrade NETs and confirm their functional role)(Papayannopoulos 2018), or a combination of both (Fig. 6K). As shown in Fig. 6L, M, both and DA and DNase I could reduce total cell and neutrophil counts in the BALF of ALI mice. Furthermore, DA or DNase I alone attenuated the LPS-induced elevation of the key neutrophil chemoattractants KC and CXCL10 in BALF (Fig. 6N; Supplementary Fig. 4G). Notably, the combined treatment did not yield a greater reduction in these parameters compared to each monotherapy, suggesting that DA and DNase I may act on overlapping pathways. Consistently, histopathological assessment revealed that both treatments individually alleviated lung injury, while their combination provided no additional protective benefit (Fig. 6O; Supplementary Fig. 4H). Collectively, our integrated data support the hypothesis that DA alleviates ALI progression by modulating macrophage-neutrophil crosstalk, thereby restraining neutrophil recruitment, inflammatory response, and NETosis.

### DA regulates NETosis by modulating the CXCL10-CXCR3 axis partly via IL-10/STAT3 signaling in macrophage-neutrophil crosstalk

**Fig. 7:**
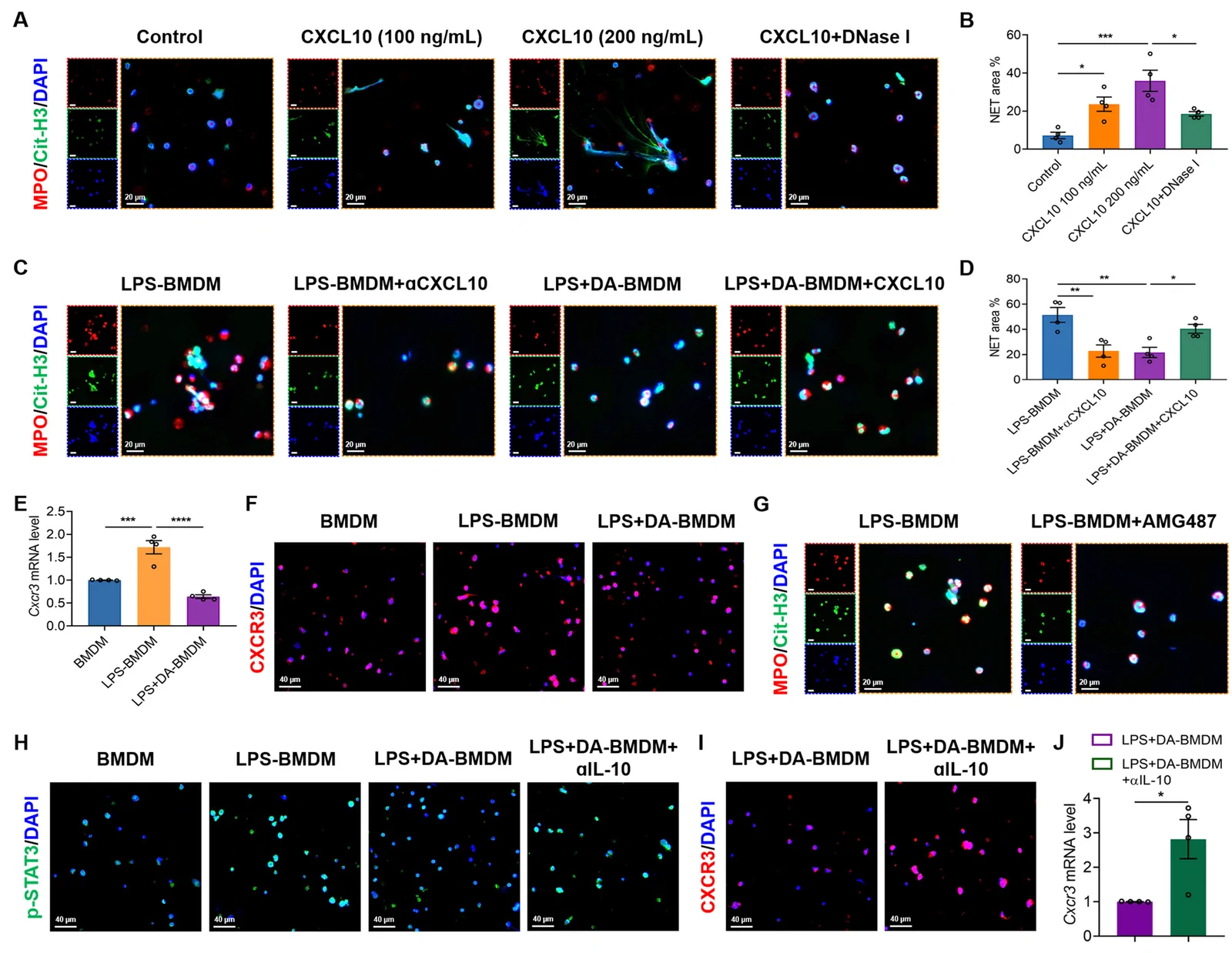
DA modulates macrophage-neutrophil crosstalk via the IL-10-CXCL10-CXCR3 axis to prevent excessive NETosis. **A**,** B** Immunofluorescence staining (**A**) and the corresponding quantification (**B**) showing NETosis markers MPO (shown in red) and Cit-H3 (shown in green) in BMDNs treated with CXCL10 with or without DNase I (*n* = 4 per group). NET‑positive area fraction = MPO⁺Cit‑H3⁺ area/total DAPI‑stained nuclear area. Nuclei were stained with DAPI, displayed in blue. Scale bar: 20 μm. **C**,** D**,** G** Immunofluorescence staining (**C**, **G**) and the corresponding quantification (**D**) showing NETosis markers MPO (shown in red) and Cit-H3 (shown in green) in BMDNs from the indirect co-culture system (*n* = 4 per group). NET‑positive area fraction = MPO⁺Cit‑H3⁺ area/total DAPI‑stained nuclear area. Nuclei were stained with DAPI, displayed in blue. Scale bar: 20 μm. **E**,** J** The *Cxcr3* mRNA level in BMDNs from the indirect co-culture system (*n* = 4 per group) Gene expression levels were normalized to *β-actin*. **F**,** I** Immunofluorescence staining exhibiting CXCR3 (shown in red) in BMDNs from the indirect co-culture system (*n* = 4 per group). Nuclei were stained with DAPI, displayed in blue. Scale bar: 40 μm. **H** Immunofluorescence staining exhibiting p-STAT3 (shown in green) in BMDNs from the indirect co-culture system (*n* = 4 per group). Nuclei were stained with DAPI, displayed in blue. Scale bar: 40 μm. All immunofluorescence staining quantification was performed in at least 6 random fields per group. Data are presented as mean ± SEM and analyzed with a 95% confidence interval. Statistical analysis was performed using two-tailed unpaired Student t test or one-way ANOVA followed by Bonferroni’s post hoc test. *p < 0.05, **p < 0.01, ***p < 0.001, ****p < 0.0001. “n” represents the number of independent biological replicates

Building on prior sequencing and ELISA data indicating DA-regulated CXCL10 expression in BALF and BMDM supernatants (Figs. 2G and 6B; Supplementary Fig. 4G, 5 A), we investigated its role in NETosis. Direct stimulation of neutrophils with recombinant CXCL10 dose-dependently exacerbated NETosis, an effect abrogated by DNase I (Fig. 7A, B). In conditioned medium co-culture experiments, neutralizing CXCL10 (αCXCL10) attenuated NETosis, whereas supplementing exogenous CXCL10 reversed DA’s suppressive effect (Fig. 7C, D). Critically, these effects were independent of cytotoxicity (Supplementary Fig. 5B, C), confirming CXCL10 as a DA-regulated mediator of NETosis. Given that CXCR3 is a primary receptor for CXCL10(Tokunaga et al. 2018), we sought to determine whether neutrophil-expressed CXCR3 is involved in this process. DA downregulated CXCR3 expression at the transcriptional level in neutrophils cultured in conditioned medium from macrophages (Fig. 7E). Consistently, immunofluorescence assays revealed a corresponding reduction in CXCR3 protein abundance (Fig. 7F; Supplementary Fig. 5D). Pharmacological inhibition of CXCR3 with AMG487 blocked LPS-induced NETosis (Fig. 7G; Supplementary Fig. 5E, F), establishing the functional necessity of the CXCL10-CXCR3 axis.

To delineate the link between CXCL10 and CXCR3, we examined the phosphorylated STAT3 (p-STAT3), a known transcriptional regulator of *Cxcr3*. Immunofluorescence showed that BMDM-derived conditioned medium following DA treatment attenuated LPS-induced p-STAT3 upregulation in BMDNs, an effect that was largely reversed by αIL-10 (Fig. 7H; Supplementary Fig. 5G). Consistently, the downregulation of p-STAT3 was accompanied by reduced CXCR3 expression: both immunofluorescence and RNA analyses confirmed that conditioned medium from DA-treated BMDMs decreased CXCR3 at the protein and mRNA levels in BMDNs, and αIL-10 treatment significantly restored its expression (Fig. 7E, F, I, J; Supplementary Fig. 5D, H). Additionally, rIL-10 not only significantly inhibited CXCL10-induced NETosis but also reduced the high expression of CXCR3 and p-STAT3 induced by conditioned medium from LPS-treated BMDMs (Supplementary Fig. 5I, J). These findings further support a causal role for IL-10 in suppressing the CXCL10-CXCR3 axis.

To extend these observations in vivo, we performed additional experiments in LPS-induced ALI mice. Exogenous CXCL10 partially reversed the protective effects of DA, as evidenced by increased BALF total cell counts, neutrophil numbers, and KC levels. AMG487 alone exhibited anti-inflammatory effects, but no additive effect was observed when combined with DA. Importantly, αIL-10 significantly attenuated DA’s anti-inflammatory effects compared with isotype control, confirming that IL-10 is functionally required in vivo (Supplementary Fig. 6). In summary, DA, via macrophage-derived IL-10, inhibits STAT3 activation to downregulate neutrophil CXCR3 expression, thereby attenuating CXCL10-CXCR3-driven NETosis and mitigating excessive inflammatory responses.

### The key rate-limiting enzyme of FAO, CPT1A in macrophage mediates DA’s protection against neutrophilic inflammation in ALI

**Fig. 8:**
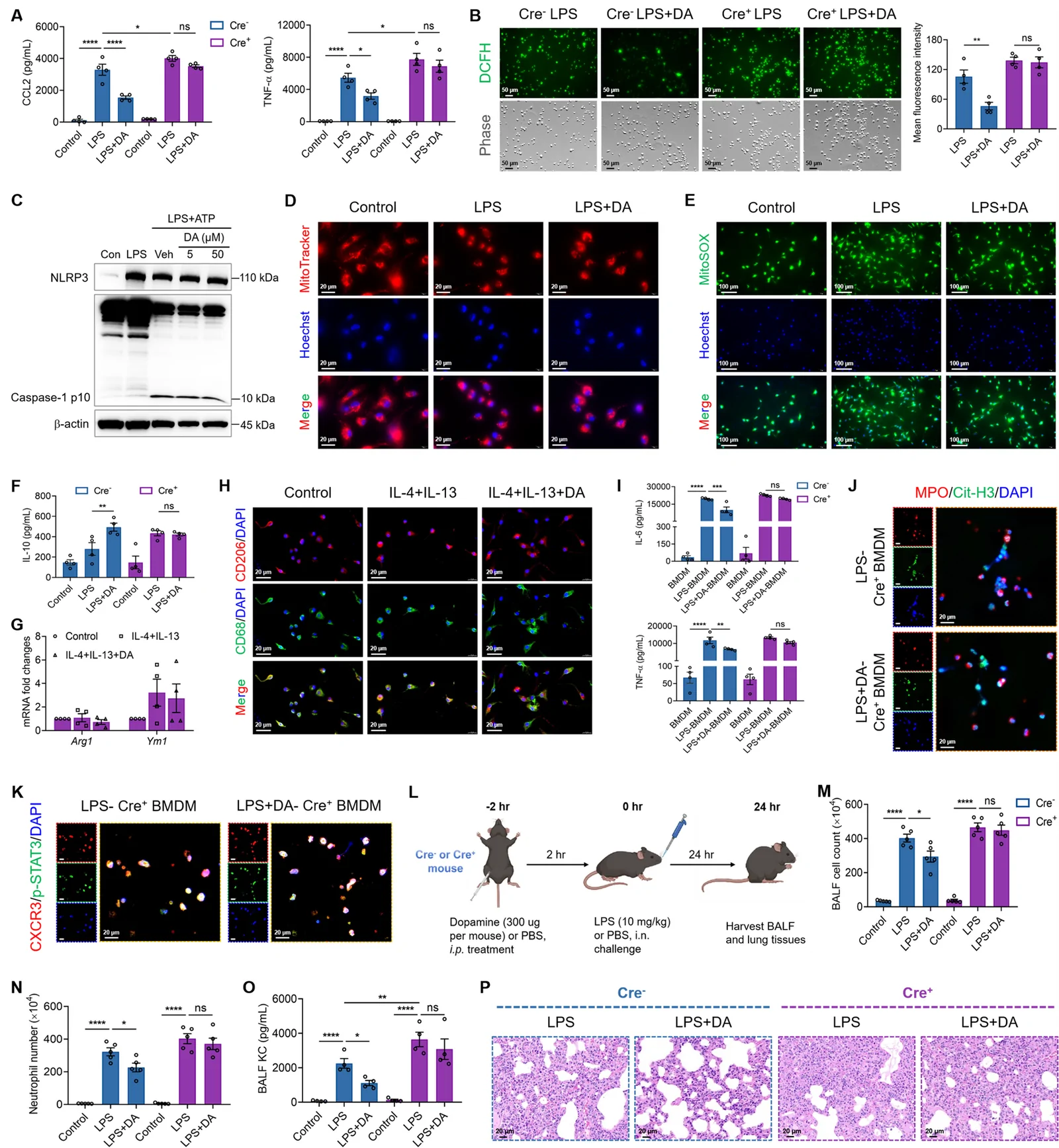
Macrophage FAO rate-limiting enzyme, CPT1A, mediates DA’s protective effects against excessive neutrophilic inflammation in ALI. **A**, **F** Secretions of CCL2, TNF-α, (**A**) and IL-10 (**F**) in Cre^−^ *Cpt1a*^fl/fl^ (Cre^−^) and Cre^+^ *Cpt1a*^fl/fl^ (Cre^+^) BMDMs treated with DA and/or LPS (*n* = 4 per group). **B** Representative images of intracellular ROS staining (DCFH-DA probe, shown in green) with corresponding brightfield views, and analysis quantified as the mean fluorescence intensity in BMDMs across treatment groups (*n* = 4 per group). Scale bar: 50 μm. **C** Immunoblotting detection of NLRP3 inflammasome activation, including NLRP3 expression and Caspase-1 activation in Cre^+^ BMDMs under indicated treatments (*n* = 3 per group). Band intensities were normalized to β-actin. **D**, **E** Representative images of Cre^+^ BMDMs stained with MitoTracker (D, shown in red) and MitoSOX (E, shown in green) probes upon different treatments (*n* = 4 per group). The nuclei were counterstained with Hoechst, shown in blue. Scale bars: 20 μm for MitoTracker probe, 100 μm for MitoSOX probe. **G** Induction of *Arg1* and *Ym1* mRNA expression in Cre⁺ BMDMs was estimated by qPCR assay (*n* = 4 per group). Gene expression levels were normalized to *β-actin*. **H** Immunofluorescence staining for M2 (CD68/CD206) markers in Cre^+^ BMDMs under various treatments (*n* = 4 per group). Nuclei were stained with DAPI, displayed in blue. Scale bar: 20 μm. **I** Levels of IL-6 and TNF-α in supernatants from the indirect co-culture system (*n* = 4 per group). **J** Immunofluorescence staining of NETosis markers MPO (shown in red) and Cit-H3 (shown in green) in BMDNs from the indirect co-culture system (*n* = 4 per group). Nuclei were stained with DAPI, displayed in blue. Scale bar: 20 μm. **K** Immunofluorescence staining of CXCR3 (shown in red) and p-STAT3 (shown in green) in BMDNs from the indirect co-culture system (*n* = 4 per group). Nuclei were stained with DAPI, displayed in blue. Scale bar: 20 μm. **L** Experimental schematic: Cre^−^ or Cre^+^ mice were pretreated with an intraperitoneal injection of DA (300 µg per mouse) or PBS 2 h prior to an intranasal challenge with LPS (10 mg/kg) or PBS. Samples were harvested 24 h post-challenge. **M**-**O** Total cell count (M, *n* = 5 per group), neutrophil number (N, *n* = 5 per group), and KC level (O, *n* = 4 per group) in BALF were quantified across groups. **P** Representative H&E-stained lung sections from different experimental groups (*n* = 5 per group). Scale bar: 20 μm. All immunofluorescence staining quantification was performed in at least 6 random fields per group. Data are presented as mean ± SEM and analyzed with a 95% confidence interval. Statistical analysis was performed using one-way ANOVA followed by Bonferroni’s post hoc test. *p < 0.05, **p < 0.01, ***p < 0.001, ****p < 0.0001, ns, not significant. “n” represents the number of independent biological replicates

Having established that DA promoted M2 polarization by enhancing FAO and maintaining mitochondrial homeostasis, we next asked whether CPT1A, the key rate-limiting enzyme of FAO(Wang et al. 2021), is required for this effect. We generated mice with a macrophage-specific knockout of *Cpt1a* (Cre⁺; validation in Supplementary Fig. 7A), which did not affect baseline cell viability (Supplementary Fig. 7B). In CPT1A-deficient BMDMs, the anti-inflammatory effects of DA were comprehensively abrogated. Specifically, DA failed to suppress LPS-induced secretion of CCL2 and TNF-α, reduce excess ROS, and inhibit NLRP3 inflammasome activation (Fig. 8A-C). Meanwhile, the ability of DA to preserve mitochondrial integrity and mitigate mitochondrial oxidative stress was also abolished by CPT1A deficiency (Fig. 8D, E; Supplementary Fig. 7C, D). Furthermore, DA’s capacity to promote M2 polarization, assessed by IL-10 secretion, *Arg1*/*Ym1* expression, and CD206 staining, was significantly abrogated by CPT1A knockout (Fig. 8F-H). Notably, the inhibitory effect of DA on LPS-elevated CXCL10 was completely abolished in CPT1A-deficient BMDMs (Supplementary Fig. 7E), implying that CPT1A acts as an essential upstream mediator in the DA-dependent modulation of macrophage-neutrophil crosstalk.

Subsequently, we investigated whether CPT1A deficiency in macrophages affects neutrophil activation. Conditioned medium from DA-treated, CPT1A-deficient BMDMs lost the capacity to suppress neutrophil inflammatory responses. Specifically, it failed to inhibit excessive secretion of IL-6 and TNF-α, block NETosis, or downregulate the p-STAT3/CXCR3 pathway in neutrophils (Fig. 8I-K; Supplementary Fig. 7G, H). These effects were independent of cytotoxicity (Supplementary Fig. 7F). Finally, in an in vivo LPS-induced ALI model, the protective effects of DA were also abolished in Cre⁺ mice compared with the control (Cre^−^) mice (Fig. 8L). DA pretreatment no longer reduced total inflammatory cell or neutrophil counts in the BALF (Fig. 8M, N), suppressed the neutrophil chemoattractant KC (Fig. 8O), or alleviated lung histopathological damage (Fig. 8P; Supplementary Fig. 7I).

To directly test whether FAO flux itself is causally required, we performed complementary pharmacological interventions. As shown in Supplementary Fig. 8, the FAO inhibitor etomoxir reversed DA’s suppression of LPS-induced pro-inflammatory cytokines and abolished DA’s promotion of IL‑10 secretion, whereas the FAO enhancer L‑carnitine enhanced these anti‑inflammatory effects. Moreover, in the co-culture system, etomoxir pretreatment of macrophages abrogated DA’s ability to suppress neutrophil NETosis, while L‑carnitine reinforced it. Collectively, these integrated in vitro and in vivo data demonstrate that macrophage CPT1A is required for the observed anti-inflammatory and FAO-promoting effects of DA, which maintain mitochondrial homeostasis, resolve macrophage inflammation, and ultimately restrain neutrophil-driven pathology in ALI.

### DA suppresses hyperinflammatory responses in human PBMCs-derived macrophages from healthy donors and ARDS patients

**Fig. 9:**
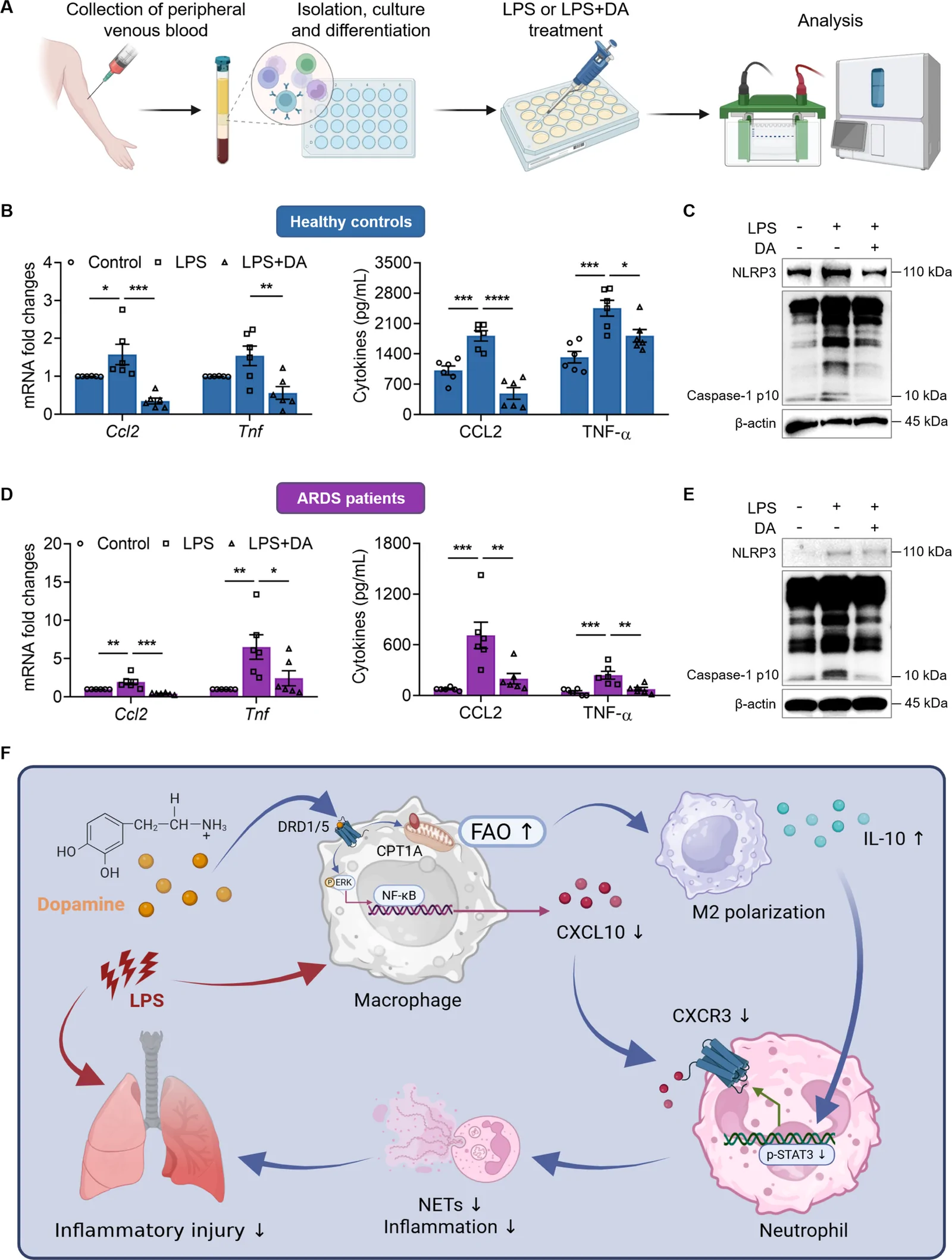
DA modulates excess inflammatory responses in peripheral blood mononuclear cells (PBMCs)-derived macrophages from healthy controls and ARDS patients. **A** Experimental schematic: human PBMCs were isolated from peripheral blood, induced differentiation into macrophages, treated with LPS with or without DA, and subjected to further analysis. **B** The mRNA and protein levels of CCL2, TNF-α in human PBMCs-derived macrophages from healthy controls upon distinct treatments (*n* = 6 per group). **C** Immunoblotting detection of NLRP3 inflammasome activation, including NLRP3 expression and Caspase-1 activation in human PBMCs-derived macrophages from healthy controls (*n* = 3 per group). **D** The mRNA and protein levels of CCL2, TNF-α in PBMCs-derived macrophages from ARDS patients upon distinct treatments (*n* = 6 per group). **E** Immunoblotting detection of NLRP3 inflammasome activation, including NLRP3 expression and Caspase-1 activation in PBMCs-derived macrophages from ARDS patients (*n* = 3 per group). **F** Schematic summarizing how DA orchestrates protection in ALI via macrophage-neutrophil crosstalk. All band intensities were normalized to β-actin, while gene expression levels were normalized to *Gapdh*. Data are presented as mean ± SEM and analyzed with a 95% confidence interval. Statistical analysis was performed using one-way ANOVA followed by Bonferroni’s post hoc test. *p < 0.05, **p < 0.01, ***p < 0.001, ****p < 0.0001. “n” represents the number of independent biological replicates

To evaluate the translational potential of DA in curbing dysregulated immune inflammation, we investigated its effects on PBMCs isolated from both healthy donors and patients with ARDS (Supplementary Table 3). PBMCs were cultured in vitro, induced to differentiate into macrophages, and subsequently treated with LPS in the presence or absence of DA (Fig. 9A; Supplementary Fig. 9A). In PBMCs-derived macrophages from healthy donors, LPS stimulation induced *Ccl2* and *Tnf* transcription, which was markedly suppressed by DA treatment. This inhibitory effect was confirmed at the protein level, as DA significantly reduced LPS-elevated secretion of CCL2 and TNF-α (Fig. 9B). Immunoblot analysis further demonstrated that DA suppressed LPS-driven activation of the NLRP3 inflammasome, evidenced by downregulation of NLRP3 and cleaved Caspase-1 (Fig. 9C).

A critical and consistent finding was observed in PBMCs-derived macrophages from ARDS patients. While quantitative analysis revealed that these cells exhibited an overall blunted inflammatory responsiveness to LPS compared to healthy donor cells, consistent with prior reports of endotoxin tolerance in ARDS(Mokart et al. 2003, Buttenschoen et al. 2008), the regulatory effect of DA was preserved. DA treatment effectively suppressed the LPS-induced cytokine production and NLRP3 inflammasome activation in patient-derived PBMCs (Fig. 9D, E). The suppressive effects of DA were not attributable to cytotoxicity, as cell viability remained unaffected by either LPS or DA in PBMCs from both cohorts (Supplementary Fig. 7B, C).

To further support the translational relevance of our findings, we examined key molecular nodes of the protective pathway in human macrophages. As shown in Supplementary Fig. 9D, E, DA treatment significantly upregulated the mRNA expression of *Cpt1a* and *Pparα*, and increased IL‑10 secretion in LPS‑stimulated human macrophages derived from healthy donors. Taken together, these data demonstrate that DA retains its capacity to temper excessive inflammatory signaling in human immune cells, even in the context of the altered immune state found in ARDS patients. These observations provide preliminary support for the preclinical translational potential of targeting dopaminergic pathways to restore immune homeostasis in clinical hyperinflammatory conditions.

## Discussion

The lung represents a quintessential site of neuro-immune integration, characterized by dense innervation and persistent immune surveillance(Baral et al. 2018, Azzoni et al. 2024). Within this complex milieu, DA emerges as a potent immunomodulator, whose effects are paradoxically context-dependent. Whether DA exerts a beneficial or detrimental role is determined by the convergence of specific receptor subtypes, cellular environments, and disease states(Beaulieu and Gainetdinov 2011). This biological plasticity stems from a fundamental pharmacological principle: the outcome of DR signaling is critically shaped by the constellation of other active pathways within the target cell. Consequently, DA can suppress B cell-mediated arthritis via D1-like receptors(Wieber et al. 2022), yet promote allergic inflammation via DRD4(Wang et al. 2019). Strikingly, DA can even exert opposing effects within the same myeloid lineage. It enhances inflammatory cytokine secretion in isolated monocyte-derived macrophages(Gaskill et al. 2012), yet dampens it within the multicellular network of PBMCs(Bergquist et al. 2000). Its role can reverse based solely on immune activation status, displaying anti-inflammatory activity in resting monocytes but shifting to a pro-inflammatory phenotype upon acute stimulation(Fleige and Capellino 2025). This spectrum of actions underscores the context-dependent nature of DA signaling. In simplified and isolated systems, a high-dose DA bolus may act as a non-specific stress signal and potentially prime pro-inflammatory responses(Nolan et al. 2020). Although the DA concentrations used in this study fall within a pharmacologically active range rather than basal physiological circulating levels, similar dosing strategies have been widely employed in experimental inflammatory models. The biological consequences of DA signaling are not solely determined by absolute concentration but are profoundly shaped by the inflammatory milieu, receptor landscape, and cellular metabolic state. In the integrated pathological network of LPS‑induced ALI, DA acts as a context‑dependent modulator, and its protective effects are mediated through specific receptor‑dependent pathways rather than non‑specific toxicity. Nevertheless, future studies should explore optimized dosing regimens and targeted delivery strategies to enhance translational potential. Our current study indicates that DA engages a coherent protective program, in which activation of D1-like receptors appears to initiate a protective cascade in macrophages. This likely involves concurrently suppressing pro-inflammatory MAPK/NF-κB/NLRP3 signaling and driving CPT1A-dependent metabolic reprogramming toward FAO and M2 polarization. The reprogrammed macrophages secrete IL-10, which contributes to inhibiting the CXCL10-CXCR3 axis, thereby restraining neutrophilic inflammation and NETosis. This reshaped microenvironment in turn reinforces macrophage programming, establishing a self-sustaining resolution circuit. Thus, in ALI, DA functions not as a simple, context-agnostic stimulant, but rather as a system-level immunometabolic regulator that harnesses and amplifies the endogenous resolution network to restore homeostasis. This work underscores a fundamental translational principle: the therapeutic logic of neuromodulators can only be fully decoded within the complete pathological tapestry, not from isolated cellular or molecular threads.

Macrophages are the initiating immune cells in LPS‑induced ALI. Their polarization state, along with distinct metabolic profiles, dictates the direction of the inflammatory response. Classically activated (M1) macrophages rely on enhanced aerobic glycolysis and impaired mitochondrial OXPHOS, and release pro‑inflammatory cytokines such as TNF‑α and IL‑6. In contrast, alternatively activated (M2) macrophages exhibit higher levels of mitochondrial OXPHOS and FAO, supporting their anti-inflammatory function characterized by IL-10 secretion(Mosser and Edwards 2008, Patel et al. 2017). This tight link between metabolic reprogramming and phenotype is exemplified in ALI, where the disease progression involves a shift from an M1 to a resolving M2 state in local macrophages(Pearce and Pearce 2013). The regulatory effect of DA on macrophage polarization is complex and appears contradictory. While one study shows it drives an M2-to-M1 switch in tumors(Qin et al. 2015), another finds it promotes M2 polarization via DRD5 signaling(Liu et al. 2021). Besides, the polymer polydopamine also induces an M1-to-M2 shift(Shen et al. 2025). However, the underlying mechanisms, particularly how DA engages cellular metabolism, are not well defined. Our study directly addresses this gap by testing the hypothesis that DA acts through mitochondrial metabolic reprogramming, specifically by enhancing FAO. We found that LPS stimulation induced M1 polarization and suppressed FAO, whereas DA reversed these effects. DA repaired LPS-induced mitochondrial structural damage, restored morphology, rebalanced fusion-fission dynamics, and upregulated the key FAO regulatory axis (PPARα-CPT1A)(Wang et al. 2021). It also restored the LPS-downregulated mRNA expression of *Cpt1a* and *Pparα*, increased the FAO-linked OCR, and concurrently reduced mitochondrial ROS overproduction. In vitro analyses showed that this metabolic reprogramming shifted macrophages from an LPS‑induced M1 toward an M2 phenotype, which is associated with reduced lung inflammation and enhanced repair(Wang et al. 2023, Wang et al. 2024). Most importantly, experiments using macrophage-specific *Cpt1a* knockout mice and pharmacological modulation of FAO demonstrated that CPT1A is required for DA to mediate these metabolic and anti‑inflammatory effects. This finding supports a core mediating role of CPT1A and provides a preliminary framework linking dopaminergic signaling, metabolic reprogramming, macrophage polarization, and protection against acute pulmonary inflammation. Nevertheless, CPT1A‑dependent FAO is likely not the sole mechanism but rather one among multiple metabolic pathways. Future studies incorporating comprehensive metabolic profiling are needed to delineate additional contributing routes.

Our study reveals an obvious paradox: DA suppresses neutrophilic inflammation in vivo but not directly in vitro, suggesting the involvement of an upstream cellular mediator. Macrophages emerge as the prime candidate, positioning the macrophage-neutrophil crosstalk as the key mechanism underlying DA’s protective effects in ALI. This crosstalk is a central determinant of inflammatory outcomes and acts as a double-edged sword in disease pathogenesis. Pyroptotic macrophages exacerbate injury by releasing microvesicles that activate neutrophils and promote NETosis(Kuang et al. 2024). Conversely, alveolar macrophages recruit neutrophils for defense(Pei et al. 2024), and specialized lung interstitial macrophages can directly suppress NETosis(Han et al. 2024). This duality makes the interface a therapeutic target, exemplified by nanoparticles that promote neutrophil apoptosis and macrophage efferocytosis to establish an anti-inflammatory cycle in ARDS(Wang et al. 2025). Such strategies confirm that modulating this cellular dialogue is feasible in complex conditions. The role of neurotransmitters in regulating this specific crosstalk, however, remains underexplored. In neuro-immunity, mediators like calcitonin gene-related peptide inhibit neutrophil and macrophage recruitment to coordinate tissue repair(Lu et al. 2024). Extending this paradigm, our work demonstrates that DA orchestrates pulmonary macrophage-neutrophil crosstalk to resolve inflammation. Mechanistically, based on indirect co-culture and pharmacological inhibition experiments, DA-reprogrammed macrophages modulate neutrophils via the CXCL10-CXCR3 axis, which acts as one of the key effectors within a broader chemokine signaling network, thereby inhibiting NETosis and excessive inflammatory responses. This hierarchical “macrophage-reprogramming to neutrophil-suppression” relationship aligns with findings by Hiroki et al.(Hiroki et al. 2025), further validating the concept. Thus, our findings suggest that DA functions not merely as an inhibitor of terminal effector cells but as an upstream regulator within the pulmonary inflammatory network, acting through hierarchical reprogramming of myeloid crosstalk to orchestrate inflammation resolution. This offers a neuro-immunological perspective for therapeutic intervention.

DA, traditionally studied for its roles in circulatory regulation and central nervous system functions, has gained increasing attention for its peripheral immunomodulatory effects, which exhibit a complex duality of both pro- and anti-inflammatory actions(Wieber et al. 2022, Wang et al. 2019, Yan et al. 2015). This complexity stems from its multifaceted nature: its effects are tightly linked to specific disease contexts, target organs, and receptor subtypes, and it rarely acts on a single immune cell type in isolation. Although preclinical evidence(Vohra et al. 2012, Cao et al. 2023), including our work, converges on its anti-inflammatory potential, clinical translation has been challenging. For instance, a relevant clinical trial (ClinicalTrials.gov: NCT00314704) demonstrated that intravenous infusion of DA not only failed to confer superior therapeutic efficacy but was also associated with significant adverse events, including arrhythmias and ischemic events(Backer et al. 2010). This lack of benefit and elevated risk profile is largely attributable to the non-selective, systemic nature of administration, which permits off-target activation of α/β-adrenergic receptors. Our integrated preclinical and clinical findings provide a foundation for more viable translational strategies. Notably, multiple practical translational hurdles remain to be addressed, including undefined safe dosing thresholds for ARDS patients, narrow therapeutic windows, and intrinsic cardiovascular risks associated with systemic DA administration. These caveats warrant rigorous consideration before clinical implementation. To overcome prior limitations, we propose the following optimized approaches: (1) utilizing unique surface markers or the inherent endocytic capacity of pulmonary macrophages to design targeted delivery systems. This would direct DA specifically to lung macrophages, avoiding systemic distribution and its associated drawbacks. (2) Developing highly selective agonists for D1-like receptors to elicit the desired anti-inflammatory response while minimizing activation of off-target receptors (e.g., α/β-adrenergic receptors), thereby improving therapeutic safety and efficacy. (3) Leveraging synergistic pathways, such as combining DA with NETosis inhibitors (e.g., DNase I, GSK484) or anti-inflammatory cytokines to enhance therapeutic outcomes in ALI/ARDS. (4) Establishing disease-stage-individualized dosing regimens to reconcile anti-inflammatory efficacy with cardiovascular safety, as standardized DA doses are unlikely to accommodate the heterogeneous nature of ARDS patient subgroups.

This study has several limitations that point to clear directions for future research. First, while the intranasal LPS-induced murine model replicates key features of infectious lung inflammation, it does not capture the etiological heterogeneity of clinical ARDS (e.g., resulting from sepsis, trauma, or viral pneumonia). The therapeutic time window of DA also remains to be systematically defined. Given the context-dependent nature of dopaminergic immunomodulation, the efficacy of DA requires further validation in more complex, clinically representative models. In addition, immune differences between mice and humans should be considered when extrapolating these preclinical findings, and further cross-species validation will be required. Second, owing to the limited availability of *Cpt1a* conditional knockout mice, both male and female animals were included in the relevant experiments, whereas only male mice were used for wild-type studies. Although all mice were age- and weight-matched with balanced sex distribution across groups to minimize bias, sex-stratified statistical analysis was not performed due to insufficient power. Future studies with larger sample sizes will be required to systematically evaluate potential sex differences in this neuro-immune regulatory axis. Besides, the effects of DA are dose-sensitive. The concentrations used here, both in vivo (300 µg/mouse) and in vitro (0.5–50 µM), require thorough pharmacokinetic and pharmacodynamic optimization for translational development. Furthermore, our clinical analyses were retrospective and exploratory in nature, based on limited peripheral blood sample data and clinical sample size. Future multi-center prospective studies with long-term follow-up are needed to establish a causal link between dopaminergic signaling and clinical outcomes in ARDS patients.

## Conclusion

This study delineates a coordinated neuro-immune regulatory axis wherein DA, primarily through the macrophage D1-like receptors, orchestrates a multi-layered anti-inflammatory program. Mining of public databases and experimental data reveals an accelerated turnover of DA during ALI, and our findings support DA as a beneficial mediator that acts through macrophages and neutrophils to confer in vivo protection in ALI mice. At the cellular level, DA reprograms macrophage metabolism by enhancing FAO and maintaining mitochondrial homeostasis in a CPT1A-dependent manner, promoting a shift toward an anti-inflammatory M2 phenotype while suppressing pro-inflammatory MAPK/NF-κB signaling and NLRP3 inflammasome activation. These modulated macrophages, in turn, regulate neutrophilic inflammation by secreting IL-10, which functions as a key upstream regulator to modulate the CXCL10-CXCR3 axis, thereby restraining neutrophil recruitment, hyperactivation, and excessive NETosis. Importantly, this protective mechanism is conserved in human PBMCs-derived macrophages from both healthy donors and ARDS patients, suggesting translational relevance. Collectively, our findings identify a previously unrecognized immunomodulatory role of DA in experimental ALI and provide a preliminary mechanistic framework for further investigation into dopaminergic signaling as a potential target to resolve dysregulated innate immunity in ALI/ARDS.

## Supplementary Information

Supplementary Material 1.

## Data Availability

The datasets used and/or analyzed during the current study are available from the corresponding author on reasonable request.

## References

[CR1] Padilla-Coreano N, Martínez-Rivera FJ. How dopamine guides our social world. Pharmacol Rev. 2025;77(5):100085.10.1016/j.pharmr.2025.10008540850269

[CR2] Mittal R, Debs LH, Patel AP, Nguyen D, Patel K, O’Connor G, et al. Neurotransmitters: The Critical Modulators Regulating Gut-Brain Axis. J Cell Physiol. 2017;232(9):2359–72. 10.1002/jcp.25518 PMC5772764 27512962

[CR3] Serio R, Zizzo MG. The multiple roles of dopamine receptor activation in the modulation of gastrointestinal motility and mucosal function. Auton Neurosci. 2023;244:103041. 10.1016/j.autneu.2022.103041 36372052

[CR4] Ciarka A, Vincent JL, van de Borne P. The effects of dopamine on the respiratory system: friend or foe? Pulm Pharmacol Ther. 2007;20(6):607–15. 10.1016/j.pupt.2006.10.011 17150392

[CR5] Qing J, Ren Y, Zhang Y, Yan M, Zhang H, Wu D, et al. Dopamine receptor D2 antagonism normalizes profibrotic macrophage-endothelial crosstalk in non-alcoholic steatohepatitis. J Hepatol. 2022;76(2):394–406. 10.1016/j.jhep.2021.09.032 34648896

[CR6] Han X, Ni J, Wu Z, Wu J, Li B, Ye X, et al. Myeloid-specific dopamine D(2) receptor signalling controls inflammation in acute pancreatitis via inhibiting M1 macrophage. Br J Pharmacol. 2020;177(13):2991–3008. 10.1111/bph.15026 PMC7280007 32060901

[CR7] Meng L, Wang M, Gao Y, Chen L, Wang K, Gao W, et al. Dopamine D1 receptor agonist alleviates acute lung injury via modulating inflammatory responses in macrophages and barrier function in airway epithelial cells. Free Radic Biol Med. 2023;202:2–16. 10.1016/j.freeradbiomed.2023.03.016 PMC10033496 36965538

[CR8] Mosser DM, Edwards JP. Exploring the full spectrum of macrophage activation. Nat Rev Immunol. 2008;8(12):958–69. 10.1038/nri2448 PMC2724991 19029990

[CR9] Wang L, Wang D, Zhang T, Ma Y, Tong X, Fan H. The role of immunometabolism in macrophage polarization and its impact on acute lung injury/acute respiratory distress syndrome. Front Immunol. 2023;14:1117548. 10.3389/fimmu.2023.1117548 PMC10067752 37020557

[CR10] Kelly B, O’Neill LA. Metabolic reprogramming in macrophages and dendritic cells in innate immunity. Cell Res. 2015;25(7):771–84. 10.1038/cr.2015.68 PMC4493277 26045163

[CR11] Pearce EL, Pearce EJ. Metabolic pathways in immune cell activation and quiescence. Immunity. 2013;38(4):633–43. 10.1016/j.immuni.2013.04.005 PMC3654249 23601682

[CR12] Wang M, Wang K, Liao X, Hu H, Chen L, Meng L, et al. Carnitine Palmitoyltransferase System: A New Target for Anti-Inflammatory and Anticancer Therapy? Front Pharmacol. 2021;12:760581. 10.3389/fphar.2021.760581 PMC8576433 34764874

[CR13] Laforge M, Elbim C, Frère C, Hémadi M, Massaad C, Nuss P, et al. Tissue damage from neutrophil-induced oxidative stress in COVID-19. Nat Rev Immunol. 2020;20(9):515–6. 10.1038/s41577-020-0407-1 PMC7388427 32728221

[CR14] Saffarzadeh M. Neutrophil Extracellular Traps as a Drug Target to Counteract Chronic and Acute Inflammation. Curr Pharm Biotechnol. 2018;19(15):1196–202. 10.2174/1389201020666190111164145 30636599

[CR15] Tokunaga R, Zhang W, Naseem M, Puccini A, Berger MD, Soni S, et al. CXCL9, CXCL10, CXCL11/CXCR3 axis for immune activation - A target for novel cancer therapy. Cancer Treat Rev. 2018;63:40–7. 10.1016/j.ctrv.2017.11.007 PMC5801162 29207310

[CR16] Lu YZ, Nayer B, Singh SK, Alshoubaki YK, Yuan E, Park AJ, et al. CGRP sensory neurons promote tissue healing via neutrophils and macrophages. Nature. 2024;628(8008):604–11. 10.1038/s41586-024-07237-y PMC11023938 38538784

[CR17] Kaur M, Singh D. Neutrophil chemotaxis caused by chronic obstructive pulmonary disease alveolar macrophages: the role of CXCL8 and the receptors CXCR1/CXCR2. J Pharmacol Exp Ther. 2013;347(1):173–80. 10.1124/jpet.112.201855 23912333

[CR18] Sookhai S, Wang JH, Winter D, Power C, Kirwan W, Redmond HP. Dopamine attenuates the chemoattractant effect of interleukin-8: a novel role in the systemic inflammatory response syndrome. Shock. 2000;14(3):295–9. 10.1097/00024382-200014030-00009 11028546

[CR19] Sookhai S, Wang JH, McCourt M, O’Connell D, Redmond HP. Dopamine induces neutrophil apoptosis through a dopamine D-1 receptor-independent mechanism. Surgery. 1999;126(2):314–22. 10455900

[CR20] Marino F, Pinoli M, Rasini E, Martini S, Luini A, Pulze L, et al. Dopaminergic inhibition of human neutrophils is exerted through D1-like receptors and affected by bacterial infection. Immunology. 2022;167(4):508–27. 10.1111/imm.13550 35897164

[CR21] Wang M, Wu D, Liao X, Hu H, Gao J, Meng L, et al. CPT1A-IL-10-mediated macrophage metabolic and phenotypic alterations ameliorate acute lung injury. Clin Transl Med. 2024;14(8):e1785. 10.1002/ctm2.1785 PMC11294017 39090662

[CR22] Vohra PK, Hoeppner LH, Sagar G, Dutta SK, Misra S, Hubmayr RD, et al. Dopamine inhibits pulmonary edema through the VEGF-VEGFR2 axis in a murine model of acute lung injury. Am J Physiol Lung Cell Mol Physiol. 2012;302(2):L185–92. 10.1152/ajplung.00274.2010 PMC3349364 22003095

[CR23] Cao Y, Li Y, Wang X, Liu S, Zhang Y, Liu G, et al. Dopamine inhibits group 2 innate lymphoid cell-driven allergic lung inflammation by dampening mitochondrial activity. Immunity. 2023;56(2):320–. – 35.e9. 10.1016/j.immuni.2022.12.017 36693372

[CR24] Matute-Bello G, Downey G, Moore BB, Groshong SD, Matthay MA, Slutsky AS, et al. An official American Thoracic Society workshop report: features and measurements of experimental acute lung injury in animals. Am J Respir Cell Mol Biol. 2011;44(5):725–38. 10.1165/rcmb.2009-0210ST PMC7328339 21531958

[CR25] Alberio T, Anchieri C, Piacentini L, Gentile G, Simmaco M, Biasin M, et al. Proteomic characterization of Jurkat T leukemic cells after dopamine stimulation: A model of circulating dopamine-sensitive cells. Biochimie. 2011;93(5):892–8. 10.1016/j.biochi.2011.01.015 21300132

[CR26] Wang B, Chen T, Li G, Jia Y, Wang J, Xue L, et al. Dopamine Alters Lipopolysaccharide-Induced Nitric Oxide Production in Microglial Cells via Activation of D1-Like Receptors. Neurochem Res. 2019;44(4):947–58. 10.1007/s11064-019-02730-7 30659504

[CR27] Tian P, Wu Q, He D, Zhao W, Luo L, Jia Z, et al. Breast cancer induces CD62L(+) Kupffer cells via DMBT1 to promote neutrophil extracellular trap formation and liver metastasis. Cell Discov. 2025;11(1):68. 10.1038/s41421-025-00819-8 PMC12343785 40796724

[CR28] Harrington JS, Ryter SW, Plataki M, Price DR, Choi AMK. Mitochondria in health, disease, and aging. Physiol Rev. 2023;103(4):2349–422. 10.1152/physrev.00058.2021 PMC10393386 37021870

[CR29] Archer SL. Mitochondrial dynamics–mitochondrial fission and fusion in human diseases. N Engl J Med. 2013;369(23):2236–51. 10.1056/NEJMra1215233 24304053

[CR30] Nomura M, Liu J, Rovira II, Gonzalez-Hurtado E, Lee J, Wolfgang MJ, et al. Fatty acid oxidation in macrophage polarization. Nat Immunol. 2016;17(3):216–7. 10.1038/ni.3366 PMC6033271 26882249

[CR31] Vats D, Mukundan L, Odegaard JI, Zhang L, Smith KL, Morel CR, et al. Oxidative metabolism and PGC-1beta attenuate macrophage-mediated inflammation. Cell Metab. 2006;4(1):13–24. 10.1016/j.cmet.2006.05.011 PMC1904486 16814729

[CR32] Papayannopoulos V. Neutrophil extracellular traps in immunity and disease. Nat Rev Immunol. 2018;18(2):134–47. 10.1038/nri.2017.105 28990587

[CR33] Mokart D, Guery BP, Bouabdallah R, Martin C, Blache J-L, Arnoulet C, et al. Deactivation of Alveolar Macrophages in Septic Neutropenic ARDS*. Chest. 2003;124(2):644–52. 10.1378/chest.124.2.644 12907555

[CR34] Buttenschoen K, Kornmann M, Berger D, Leder G, Beger HG, Vasilescu C. Endotoxemia and endotoxin tolerance in patients with ARDS. Langenbecks Arch Surg. 2008;393(4):473–8. 10.1007/s00423-008-0317-3 18320210

[CR35] Baral P, Umans BD, Li L, Wallrapp A, Bist M, Kirschbaum T, et al. Nociceptor sensory neurons suppress neutrophil and γδ T cell responses in bacterial lung infections and lethal pneumonia. Nat Med. 2018;24(4):417–26. 10.1038/nm.4501 PMC6263165 29505031

[CR36] Azzoni R, Perdijk O, Harris NL, Marsland BJ. Neuroimmunology of the Lung. Annu Rev Immunol. 2024;42(1):57–81. 10.1146/annurev-immunol-083122-042512 37989144

[CR37] Beaulieu JM, Gainetdinov RR. The physiology, signaling, and pharmacology of dopamine receptors. Pharmacol Rev. 2011;63(1):182–217. 10.1124/pr.110.002642 21303898

[CR38] Wieber K, Fleige L, Tsiami S, Reinders J, Braun J, Baraliakos X, et al. Dopamine receptor 1 expressing B cells exert a proinflammatory role in female patients with rheumatoid arthritis. Sci Rep. 2022;12(1):5985. 10.1038/s41598-022-09891-6 PMC8993840 35396380

[CR39] Wang W, Cohen JA, Wallrapp A, Trieu KG, Barrios J, Shao F, et al. Age-Related Dopaminergic Innervation Augments T Helper 2-Type Allergic Inflammation in the Postnatal Lung. Immunity. 2019;51(6):1102–e187. 10.1016/j.immuni.2019.10.002 PMC6937208 31757673

[CR40] Gaskill PJ, Carvallo L, Eugenin EA, Berman JW. Characterization and function of the human macrophage dopaminergic system: implications for CNS disease and drug abuse. J Neuroinflammation. 2012;9:203. 10.1186/1742-2094-9-203 PMC3488577 22901451

[CR41] Bergquist J, Ohlsson B, Tarkowski A. Nuclear factor-kappa B is involved in the catecholaminergic suppression of immunocompetent cells. Ann N Y Acad Sci. 2000;917:281–9. 10.1111/j.1749-6632.2000.tb05394.x 11268354

[CR42] Fleige L, Capellino S. Acute stimulation of PBMCs drives switch from dopamine-induced anti- to proinflammatory phenotype of monocytes only in women. Biol Sex Differ. 2025;16(1):8. 10.1186/s13293-025-00689-5 PMC11789415 39901238

[CR43] Nolan RA, Reeb KL, Rong Y, Matt SM, Johnson HS, Runner K, et al. Dopamine activates NF-κB and primes the NLRP3 inflammasome in primary human macrophages. Brain Behav Immun Health. 2020;2:100030. 10.1016/j.bbih.2019.100030 PMC7929492 33665636

[CR44] Patel U, Rajasingh S, Samanta S, Cao T, Dawn B, Rajasingh J. Macrophage polarization in response to epigenetic modifiers during infection and inflammation. Drug Discov Today. 2017;22(1):186–93. 10.1016/j.drudis.2016.08.006 PMC5226865 27554801

[CR45] Qin T, Wang C, Chen X, Duan C, Zhang X, Zhang J, et al. Dopamine induces growth inhibition and vascular normalization through reprogramming M2-polarized macrophages in rat C6 glioma. Toxicol Appl Pharmacol. 2015;286(2):112–23. 10.1016/j.taap.2015.03.021 25818600

[CR46] Liu L, Wu Y, Wang B, Jiang Y, Lin L, Li X, et al. DA-DRD5 signaling controls colitis by regulating colonic M1/M2 macrophage polarization. Cell Death Dis. 2021;12(6):500. 10.1038/s41419-021-03778-6 PMC8129081 34001860

[CR47] Shen L, Fu S, Li W, Yu K, Ran Y, Shi G, et al. Biomimetic polydopamine nanoassembly regulates M2 macrophage polarization to alleviate radiation-induced pulmonary fibrosis via the PI3K/AKT/S100A4 pathway. Chem Eng J. 2025;506:159980.

[CR48] Kuang L, Wu Y, Shu J, Yang J, Zhou H, Huang X. Pyroptotic Macrophage-Derived Microvesicles Accelerate Formation of Neutrophil Extracellular Traps via GSDMD-N-expressing Mitochondrial Transfer during Sepsis. Int J Biol Sci. 2024;20(2):733–50. 10.7150/ijbs.87646 PMC10758106 38169726

[CR49] Pei X, Liu L, Wang J, Guo C, Li Q, Li J, et al. Exosomal secreted SCIMP regulates communication between macrophages and neutrophils in pneumonia. Nat Commun. 2024;15(1):691. 10.1038/s41467-024-44714-4 PMC10805922 38263143

[CR50] Han S, Kim B, Hyeon DY, Jeong D, Ryu J, Nam JS, et al. Distinctive CD39(+)CD9(+) lung interstitial macrophages suppress IL-23/Th17-mediated neutrophilic asthma by inhibiting NETosis. Nat Commun. 2024;15(1):8628. 10.1038/s41467-024-53038-2 PMC11452667 39366998

[CR51] Wang Y, Zhang LF, Zhang JJ, Yu SS, Li WL, Zhou TJ, et al. Spontaneous Inflammation Resolution Inspired Nanoparticles Promote Neutrophil Apoptosis and Macrophage Efferocytosis for Acute Respiratory Distress Syndrome Treatment. Adv Healthc Mater. 2025;14(6):e2402421. 10.1002/adhm.202402421 39723664

[CR52] Hiroki CH, Hassanabad MF, Defaye M, Sarden N, Bartlett A, Farias R et al. Nociceptor neurons suppress alveolar macrophage-induced Siglec-F(+) neutrophil-mediated inflammation to protect against pulmonary fibrosis. Immunity. 2025;58(8):2054-68.e6. 10.1016/j.immuni.2025.05.002 40449484

[CR53] Yan Y, Jiang W, Liu L, Wang X, Ding C, Tian Z, et al. Dopamine controls systemic inflammation through inhibition of NLRP3 inflammasome. Cell. 2015;160(1–2):62–73. 10.1016/j.cell.2014.11.047 25594175

[CR54] De Backer D, Biston P, Devriendt J, Madl C, Chochrad D, Aldecoa C, et al. Comparison of dopamine and norepinephrine in the treatment of shock. N Engl J Med. 2010;362(9):779–89. 10.1056/NEJMoa0907118 20200382

