# Supplementary material for: Dopamine signaling reprograms macrophage FAO to alleviate acute lung injury by inhibiting NETosis via the CXCL10-CXCR3 axis
Source: Mol Med. 2026 Jul 4;32:162. doi: 10.1186/s10020-026-01549-7 (PMC13617839; doi:10.1186/s10020-026-01549-7)
Supplement: Supplementary file 1 [file 10020_2026_1549_MOESM1_ESM.pdf]

Supplementary Materials for

Dopamine Signaling Reprograms Macrophage FAO to Alleviate Acute Lung Injury by Inhibiting NETosis via the CXCL10-CXCR3 Axis

Methods

Cell viability detection

Cells were plated in 96-well plates at 5×10^4 cells/well and treated with LPS, DA, or other experimental agents as required. The medium was removed post-treatment, and cells were washed with 100-μL PBS, after which the PBS was discarded. A 10-fold dilution of CCK-8 reagent (Dojindo Laboratories, Japan) was added to each well, followed by a 90-120 min incubation period. Absorbance (Abs.) at 450 nm was measured with a microplate reader, and cell viability percentage was calculated using the equation: Cell viability (%) = [(Abs. (sample) - Abs. (blank) / (Abs. (control) - Abs. (blank)] × 100%.

Immunofluorescence assay

Bone marrow-derived primary macrophages (BMDMs) or bone marrow-derived primary neutrophils (BMDNs) were plated onto cell climbing slides and treated under distinct experimental conditions at for 24 h or 12 h. Post-treatment, adherent cells were fixed with 4% paraformaldehyde for 15 min at room temperature, permeabilized with 0.1% Triton X-100 for 10 min, and blocked with 5% bovine serum albumin for 1 h. Slides were incubated overnight at 4°C with primary antibodies against CD68 (Servicebio, GB113109, 1:200, China), CD86 (Proteintech, 13395-1-AP, 1:300, USA), and CD206 (Servicebio, GB113497, 1:500, China) for BMDMs; while CXCR3 (Zenbio, R381907, 1:200, China) and p-STAT3 (Zenbio, 310019, 1:200, China) for BMDNs.

After three PBS washes, slides were incubated for 2 h in the dark with secondary antibodies (goat anti-rabbit IgG-Cy3, Servicebio, GB21303, 1:300, China; goat anti-rat IgG-FITC, Servicebio, GB22302, 1:100, China) diluted in blocking buffer containing DAPI. Subsequently, stained cells were mounted with ProLong Antifade Mountant (ThermoFisher, USA) and cured overnight at room temperature in the dark. Fluorescent images were acquired using a Nikon Eclipse C1 fluorescence microscope (Nikon Corporation, Japan) equipped with a DS-U3 digital imaging system.

Immunoblotting detection

After different treatments, cells or supernatant samples were lysed with RIPA lysis buffer supplemented with protease and phosphatase inhibitors (Beyotime, China). The extracted proteins were quantified using a BCA protein assay kit (Beyotime, China). Protein samples were denatured by boiling at 95°C for 15 min, then loaded onto the gel alongside a protein marker. Electrophoresis was run at 80 V until the main bands entered the separation gel, then switched to 130 V until completion. Subsequently, proteins were transferred onto a 0.45-μm polyvinylidene difluoride membrane (Millipore, USA) at 300 mA for approximately 100 min, with the duration adjusted by the molecular weight of the target proteins. After transfer, the membrane was rinsed with Tris-buffered saline containing Tween 20 (TBST) for 5 min, blocked with 5% skimmed milk solution for 1 h, and washed three times with TBST (5 min per wash). Then, the membrane was incubated overnight at 4°C with primary antibodies against the target proteins, including TH (Zen-Bioscience, R381111, 1:1000, China), DDC

(CST, 13561, 1:1000, USA), DRD1 (Abcam, ab256555, 1:1000, UK), DRD5 (Abcam, ab181623, 1:1000, UK), β-actin (CST, 4970, 1:1000, USA), NLRP3 (CST, 15101, 1:1000, USA), Caspase-1 p10 (Abcam, ab179515, 1:1000, UK), IL-1β (CST, 12242, 1:1000, USA), p-P65 (CST, 3033, 1:1000, USA), p65 (CST, 8242, 1:1000, USA), p-ERK1/2 (CST, 4370, 1:2000, USA), ERK (CST, 4695, 1:1000, USA), p-p38 (CST, 4511, 1:1000, USA), p38 (CST, 9212, 1:1000, USA), OPA1 (CST, 80471, 1:1000, USA), p-DRP1 (CST, 4494, 1:1000, USA), Cit-H3 (Zenbio, R24572, 1:1000, China), CPT1A (Abcam, ab234111, 1:1000, UK). On the following day, the membrane was washed three times with TBST, incubated with horseradish peroxidase-conjugated secondary antibodies, either anti-rabbit or anti-mouse IgG (CST, 7074 or 7076; 1:2000, USA) for 1.5 h, and washed three times again with TBST. Finally, bands were visualized via the Tanon 5200 multifunctional chemiluminescence imaging system (Tanon Biotechnology, China), with image analysis performed using ImageJ software. The uncropped immunoblot images corresponding to Figure 1F, Figure 4F, J, Figure 5B, Figure 6F, J, Figure 8C, and Figure 9C, E were supplied in **Supplementary Figure 10**.

Enzyme-linked immunosorbent assay (ELISA)

Culture supernatants of BMDMs, BMDNs, or human peripheral blood mononuclear cells (PBMCs)-derived macrophages under different stimulatory conditions were collected and centrifuged at 2000×g for 10 min at 4 °C to thoroughly eliminate cellular debris and contaminant. ELISA was then conducted following the kit instructions with

the respective reagents: mouse CCL2 kit (MultiSciences Biotech, EK287, China), mouse TNF-α kit (MultiSciences Biotech, EK282, China), mouse IL-6 kit (MultiSciences Biotech, EK206, China), mouse CXCL10 kit (MultiSciences Biotech, EK268, China), mouse IL-10 kit (MultiSciences Biotech, EK210, China), human IL-6 kit (MultiSciences Biotech, EK106, China), human TNF-α kit (MultiSciences Biotech, EK182, China), and human IL-10 kit (MultiSciences Biotech, EK110, China).

RNA extraction and reverse transcription

After different treatments, culture medium was removed and cells were rinsed with PBS to eliminate residual medium. Trizol reagent (Invitrogen, USA) was added in an appropriate volume, and cells were lysed by pipetting repeatedly before transferring the lysate to a sterile EP tube. Chloroform was added, the mixture was vortexed vigorously, and left standing at room temperature for 3 min. Following centrifugation at 14,000 rpm for 15 min, the upper aqueous phase was collected, mixed with isopropanol, and incubated at room temperature. A second centrifugation at 14,000 rpm for 10 min yielded an RNA pellet, which was washed with 75% ethanol, centrifuged, and air-dried. The RNA was dissolved in DEPC-treated water, and reverse transcription was carried out with Takara reverse transcription Mix (Japan) under the conditions: 37°C for 15 min and 85°C for 5 s. The obtained cDNA purity was detected by spectrophotometry, and the concentration was adjusted to 500 ng/mL for downstream applications.

Quantitative polymerase chain reaction (qPCR)

A 10-μL qPCR reaction system was prepared via TB Green Premix Ex Taq^{TM} (Takara, Japan) and target gene specific primers. The mixtures were loaded onto the qPCR instrument, with relevant cycling parameters set before running the assay. The qPCR procedure (heating and cooling at 4°C/s) included pre-incubation at 50°C for 2 min and 95°C for 30 s, amplification cycles of 95°C for 5 s and 60°C for 34 s, and a melting curve stage (95°C for 15 s, 60°C for 1 min, 95°C for 1 s with a 0.2 °C/s heating rate). Relative gene expression was quantified via the $2^{-\Delta\Delta Ct}$ method. Specific primers were purchased from BioTNT (China), and their sequences are listed as follows:

Supplementary Table 1. List of qPCR primers used in this study.

Primer	Sequence	Species	NCBI Gene Accession No.
Cpt1a forward	TCCAGTTGGCTTATCGTGGTG	Mouse	NM_013495.2
Cpt1a reverse	TCCAGAGTCCGATTGATTTTTGC	Mouse	NM_013495.2
Ppara forward	GCAGCTCGTACAGGTCATCA	Mouse	NM_011144.4
Ppara reverse	CTCTTCATCCCCAAGCGTAG	Mouse	NM_011144.4
Inos (Nos2) forward	TGCCACGGACGAGACGGATAG	Mouse	NM_010927.4
Inos (Nos2) reverse	CTCTTCAAGCACCTCCAGGAACG	Mouse	NM_010927.4
Il12 (Il12a) forward	CAGAAAGGTGCGTTCCTCGTA	Mouse	NM_008351.3
Il12 (Il12a) reverse	GCCCCTTTGCATTGG	Mouse	NM_008351.3
Arg1 forward	CTCCAAGCCAAAGTCCTTAGAG	Mouse	NM_007482.3
Arg1 reverse	AGGAGCTGTCATTAGGGACATC	Mouse	NM_007482.3
Ym1 (Chil3) forward	AGAAGGGAGTTTCAAACCTGGT	Mouse	NM_009892.2
Ym1 (Chil3) reverse	GTCTTGCTCATGTGTGTAAGTGA	Mouse	NM_009892.2

Cxcr3 forward	CAGCCTGAACTTTGACAGAACCT	Mouse	NM_009910.3
Cxcr3 reverse	GCAGCCCCAGCAAGAAGA	Mouse	
β-actin forward	AACAGTCCGCCTAGAAGCAC	Mouse	NM_007393.5
β-actin reverse	CGTTGACATCCGTAAAGACC	Mouse	
Ccl2 forward	TTCTGTGCCTGCTGCTCAT	Human	NM_002982.4
Ccl2 reverse	GGGGCATTGATTGCATCT	Human	
Tnf forward	CCTCTCTCTAATCAGCCCTCTG	Human	NM_000594.4
Tnf reverse	GAGGACCTGGGAGTAGATGAG	Human	
Cpt1a forward	GATTTTGCTGTCGGTCTTGG	Human	NM_001876.4
Cpt1a reverse	CTCTTGCTGCCTGAATGTGA	Human	
Pparα forward	CGTGCTTCCTGCTTCATAGATAAG	Human	NM_001001928.3
Pparα reverse	GTGGTAGCGCTGGTCTAC	Human	
Gapdh forward	GGACTCATGACCACAGTCCA	Human	NM_002046.4
Gapdh reverse	TCAGCTCAGGGATGACCTTG	Human	

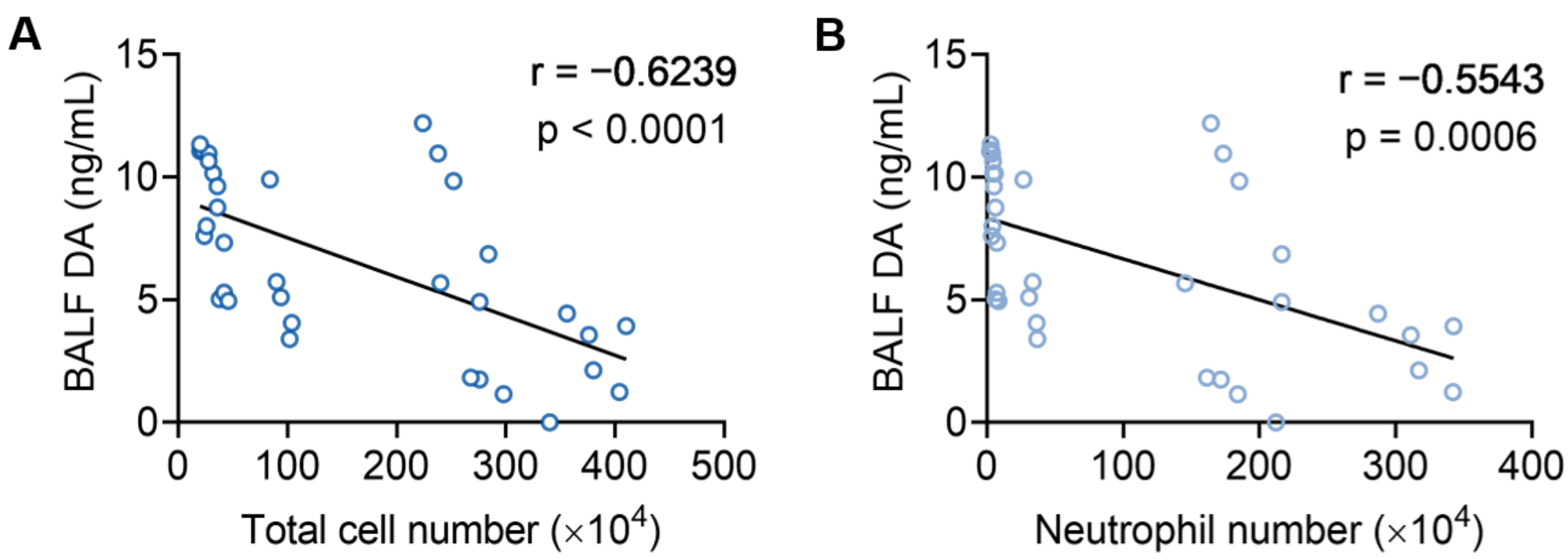

Supplementary Figure 1. (A, B) Correlation of bronchoalveolar lavage fluid (BALF) dopamine (DA) concentration with total cell number (A) and neutrophil number (B) in lipopolysaccharide (LPS)-induced acute lung injury (ALI) mice. Statistical analysis was performed via Pearson's correlation test.

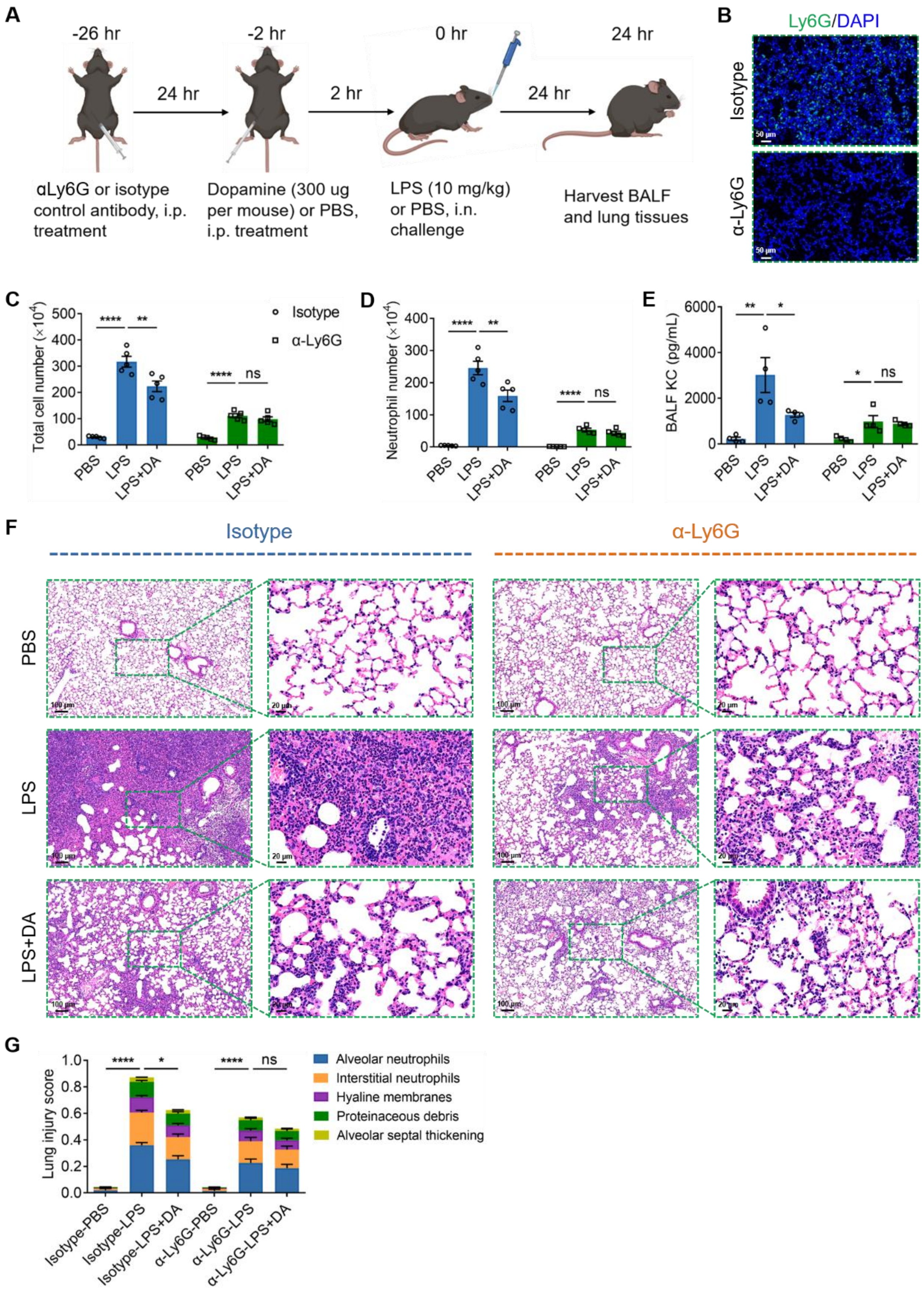

Supplementary Figure 2. (A) Experimental schematic: to deplete neutrophils, mice received an intraperitoneal administration of αLy6G or isotype control antibody (300 μg per mouse) 26 h prior to LPS challenge. Mice were then treated with an intraperitoneal injection of DA (300 μg per mouse) or PBS 2 h before the intranasal

instillation of LPS (10 mg/kg) or PBS. Samples were harvested 24 hours post-LPS challenge. **(B)** Representative immunofluorescence staining of lung tissues for Ly6G (shown in green) (n = 4 per group). Nuclei were stained with DAPI, displayed in blue. Scale bar: 50 μm. **(C-E)** Total cell count (C, n = 5 per group), neutrophil number (D, n = 5 per group), and KC level (E, n = 4 per group) in BALF were quantified across groups. **(F-G)** Representative hematoxylin and eosin (H&E)-stained lung tissue sections (F) and corresponding injury scoring (G) at 24 h post-LPS challenge (n = 5 per group). Scale bar: 100 μm in the left panel, 20 μm in the right panel. Data are presented as mean ± SEM and analyzed with a 95% confidence interval. Statistical analysis was performed using one-way ANOVA followed by Bonferroni's post hoc test. $*p < 0.05$, $**p < 0.01$, $****p < 0.0001$, ns, not significant. "n" represents the number of independent biological replicates.

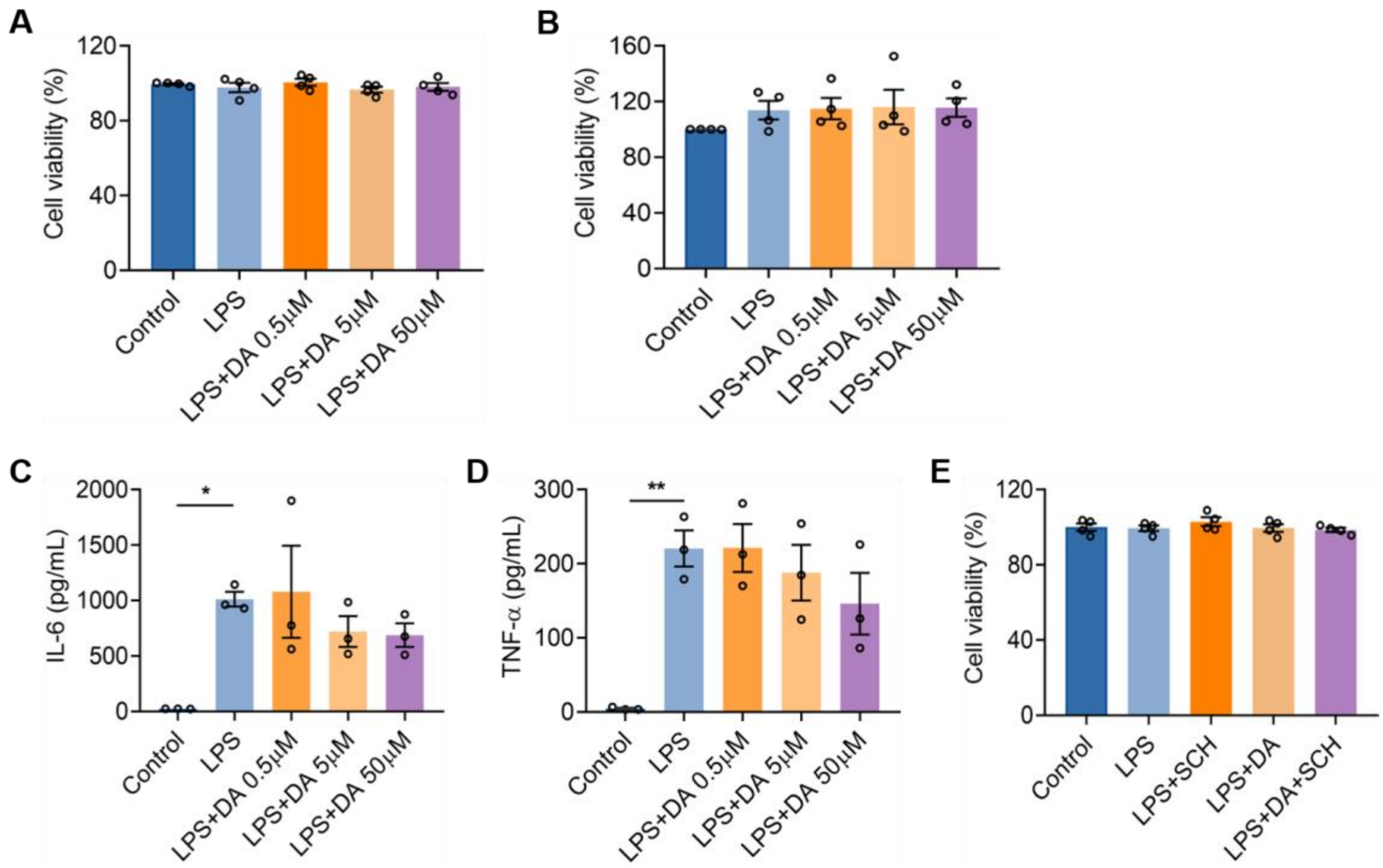

Supplementary Figure 3. (A, B) The viability of bone marrow-derived macrophages (BMDMs) (A) or bone marrow-derived neutrophils (BMDNs) (B) was assessed following treatment with various concentrations of DA (n = 4 per group). **(C, D)** Concentrations of IL-6 (C) and TNF-α (D) were detected in the culture supernatants of BMDNs under indicated treatments (n = 3 per group). **(E)** The viability of BMDMs was assessed following treatment with DA and/or SCH (n = 4 per group). Data are presented as mean ± SEM and analyzed with a 95% confidence interval. Statistical analysis was performed using one-way ANOVA followed by Bonferroni's post hoc test. *$p < 0.05$, **$p < 0.01$. "n" represents the number of independent biological replicates.

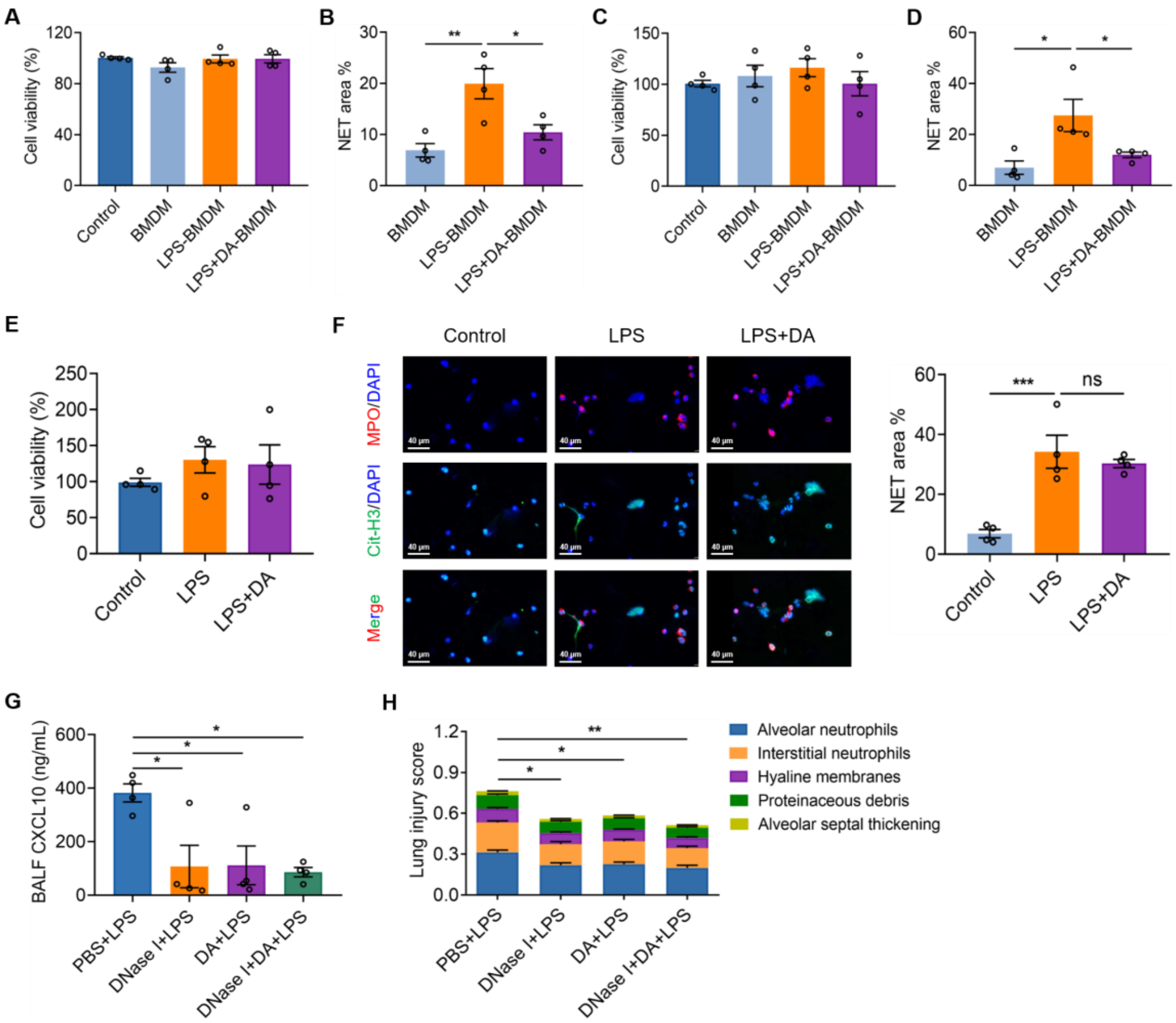

Supplementary Figure 4. (A) The viability of BMDNs treated with conditioned medium from BMDMs under different treatments (indirect co-culture system) (n = 4 per group). **(B)** Quantification of neutrophil extracellular traps (NETs) area in BMDNs from the indirect co-culture system (n = 4 per group). **(C)** The viability of BMDNs co-cultured with BMDMs under different treatments (direct co-culture system) (n = 4 per group). **(D)** Quantification of NETs area in BMDNs from the direct co-culture system (n = 4 per group). **(E)** The viability of BMDNs following distinct treatments (n = 4 per group). **(F)** Representative immunofluorescence images and the quantification of NETosis in BMDNs under distinct treatments (n = 4 per group). NET-positive area fraction = MPO$^+$Cit-H3$^+$ (double-positive for myeloperoxidase and citrullinated histone

H3) area/total DAPI-stained nuclear area. Nuclei were stained with DAPI, displayed in blue. Scale bar: 40 µm. **(G)** C-X-C motif chemokine ligand (CXCL)-10 level in BALF from mice across groups (n = 4 per group). **(H)** Lung injury score analysis of mice across groups (n = 5 per group). Data are presented as mean ± SEM and analyzed with a 95% confidence interval. Statistical analysis was performed using one-way ANOVA followed by Bonferroni's post hoc test. $^{*}p < 0.05$, $^{**}p < 0.01$, $^{***}p < 0.001$, ns, not significant. "n" represents the number of independent biological replicates.

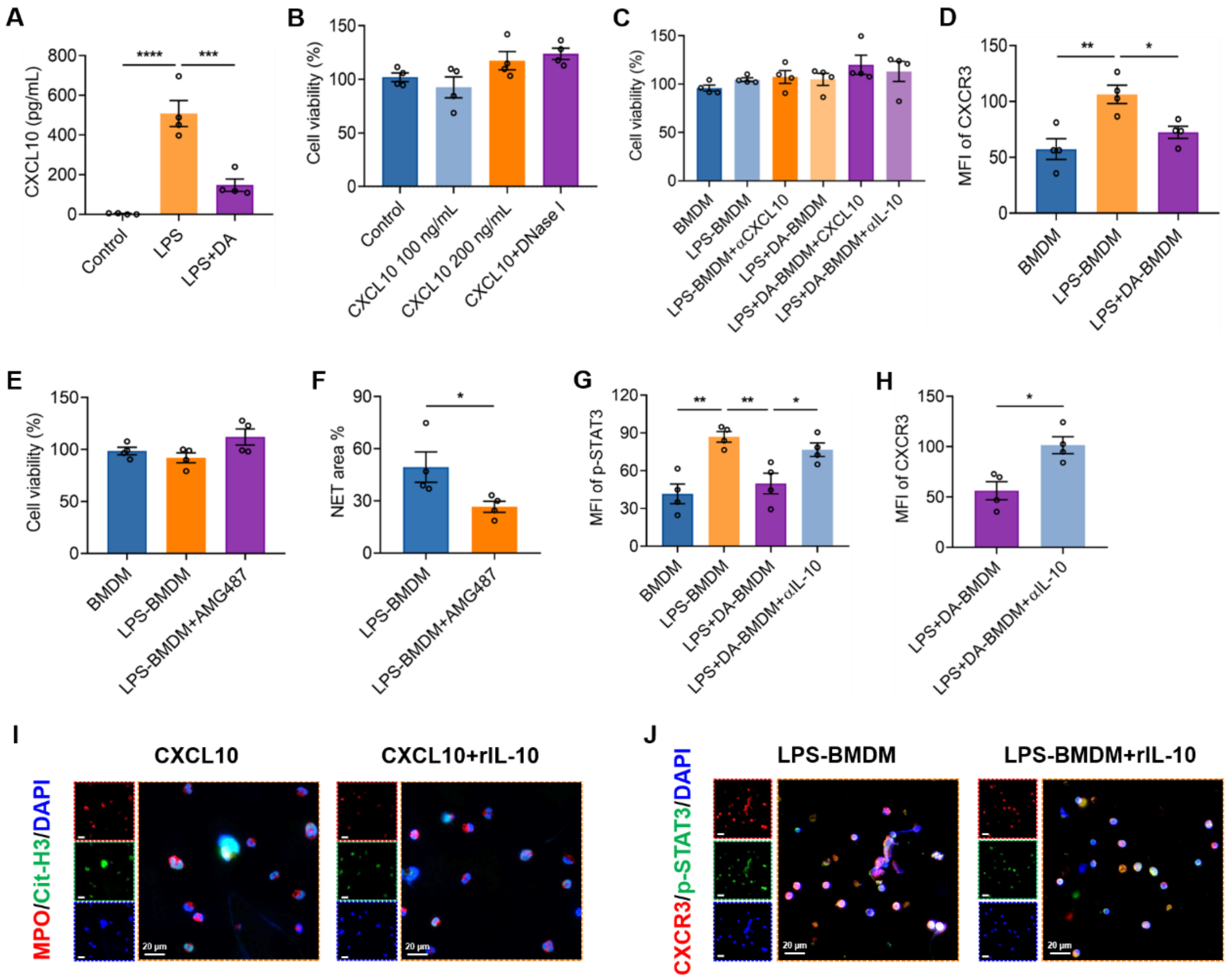

Supplementary Figure 5. (A) Concentration of CXCL10 in the culture supernatants of BMDMs under indicated treatments (n = 4 per group). **(B, C, E)** The viability of BMDNs treated with direct stimulations (B) or conditioned medium (C, E) from BMDMs under different treatments (n = 4 per group). **(D, G, H)** Quantitative analysis (mean fluorescence intensity, MFI) of CXCR3 (D, H) and p-STAT3 (G) immunofluorescence in BMDNs from the indirect co-culture system (n = 4 per group). **(F)** Quantification of NETs area in BMDNs from the indirect co-culture system (n = 4 per group). **(I)** Representative immunofluorescence images of NETosis in BMDNs under distinct treatments (n = 4 per group). Nuclei were stained with DAPI, displayed in blue. Scale bar: 20 μm. **(J)** Immunofluorescence staining of CXCR3 (shown in red) and p-STAT3 (shown in green) in BMDNs from groups (n = 4 per group). Nuclei were

stained with DAPI, displayed in blue. Scale bar: 20 μm. Data are presented as mean ± SEM and analyzed with a 95% confidence interval. Statistical analysis was performed using two-tailed unpaired Student t test or one-way ANOVA followed by Bonferroni's post hoc test. *p < 0.05, **p < 0.01, ***p < 0.001, ****p < 0.0001. "n" represents the number of independent biological replicates.

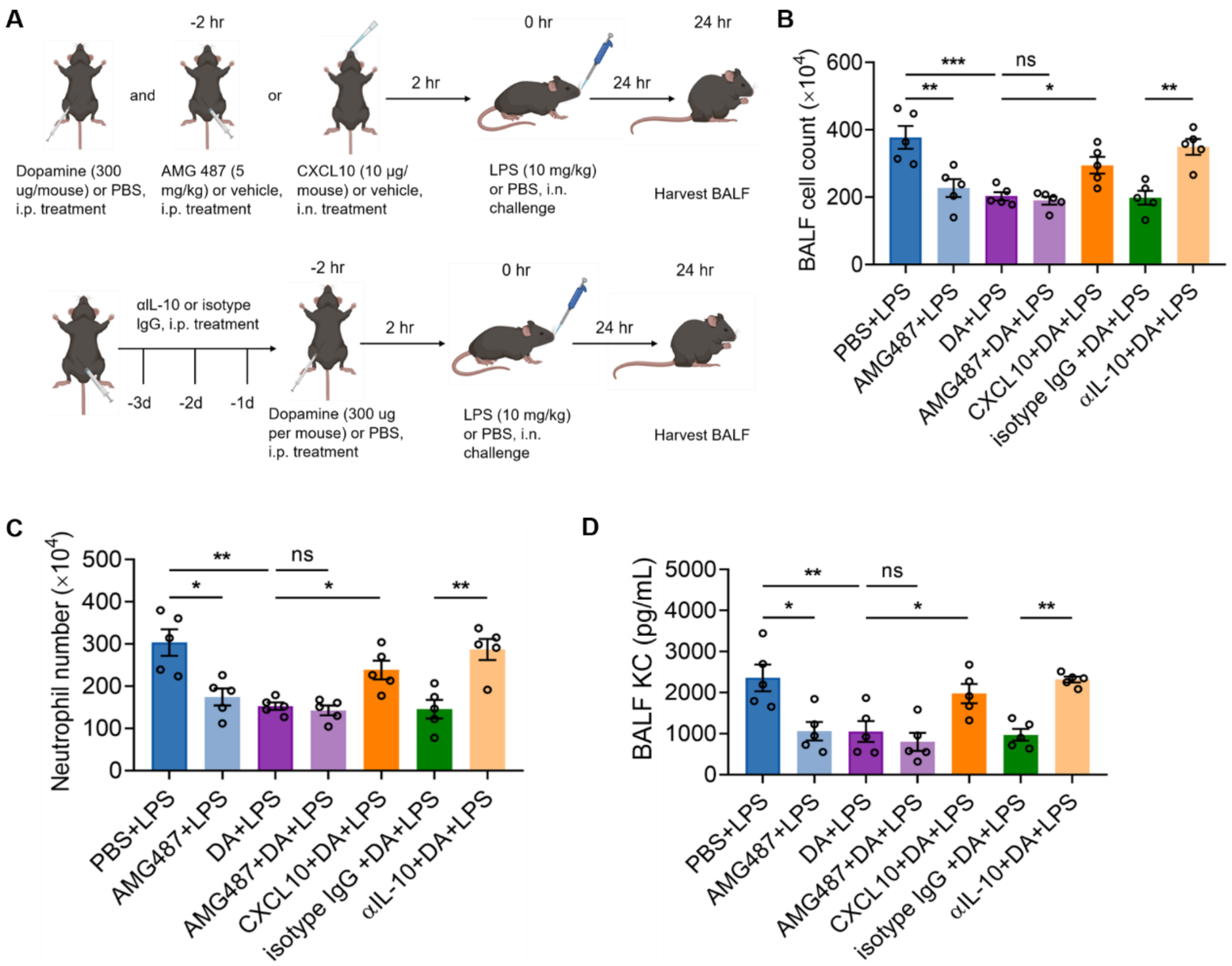

Supplementary Figure 6. (A) Experimental schematic: DA (300 μg per mouse intraperitoneally), AMG487 (5 mg/kg intraperitoneally) or recombinant CXCL10 (10 μg per mouse intranasally) was administered 2 h prior to LPS stimulation. To neutralize IL-10, mice were intraperitoneally injected with αIL-10 or its isotype control for 3 consecutive days prior to LPS challenge, at a dose of 350 μg per mouse once daily. Samples were harvested 24 hours post-LPS challenge. **(B-D)** Total cell count (B, n = 5 per group), neutrophil number (C, n = 5 per group), and KC level (D, n = 4 per group) in BALF were quantified across groups. Data are presented as mean ± SEM and analyzed with a 95% confidence interval. Statistical analysis was performed using one-way ANOVA followed by Bonferroni's post hoc test. *$p < 0.05$, **$p < 0.01$, ***$p < 0.001$, ns, not significant. "n" represents the number of independent biological replicates.

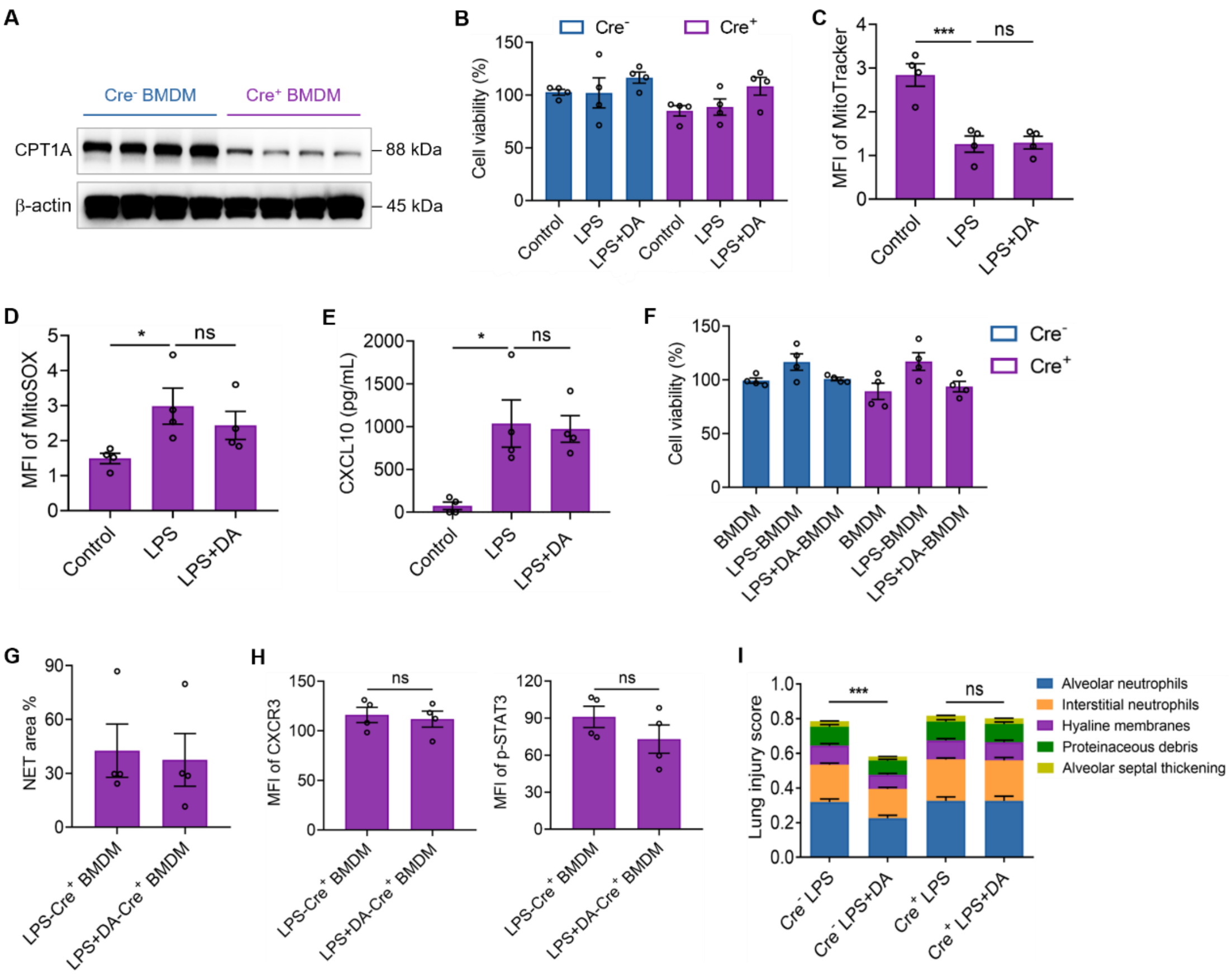

Supplementary Figure 7. (A) Immunoblot analysis of CPT1A in BMDMs from Cre⁻ and Cre⁺ mice. **(B)** The viability of BMDMs form Cre⁻ or Cre⁺ mice with different treatments (n = 4 per group). **(C, D)** Quantitative analysis of Cre⁺ BMDMs stained with MitoTracker (C) and MitoSOX (D) probes upon indicated treatments (n = 4 per group). **(E)** Concentration of CXCL10 in the culture supernatants of Cre⁺ BMDMs under distinct treatments (n = 4 per group). **(F)** The viability of BMDNs treated with conditioned medium from Cre⁻ and Cre⁺ BMDMs upon different treatments (n = 4 per group). **(G, H)** Quantification of NETs area (G) and CXCR3, p-STAT3 immunofluorescence (H) in BMDNs treated with conditioned medium from Cre⁺ BMDMs under distinct treatments (n = 4 per group). **(I)** Lung injury score analysis of mice across groups (n = 5 per group). Data are presented as mean ± SEM and analyzed

with a 95% confidence interval. Statistical analysis was performed using two-tailed unpaired Student t test or one-way ANOVA followed by Bonferroni's post hoc test. *$p < 0.05$, ***$p < 0.001$, ns, not significant. "n" represents the number of independent biological replicates.

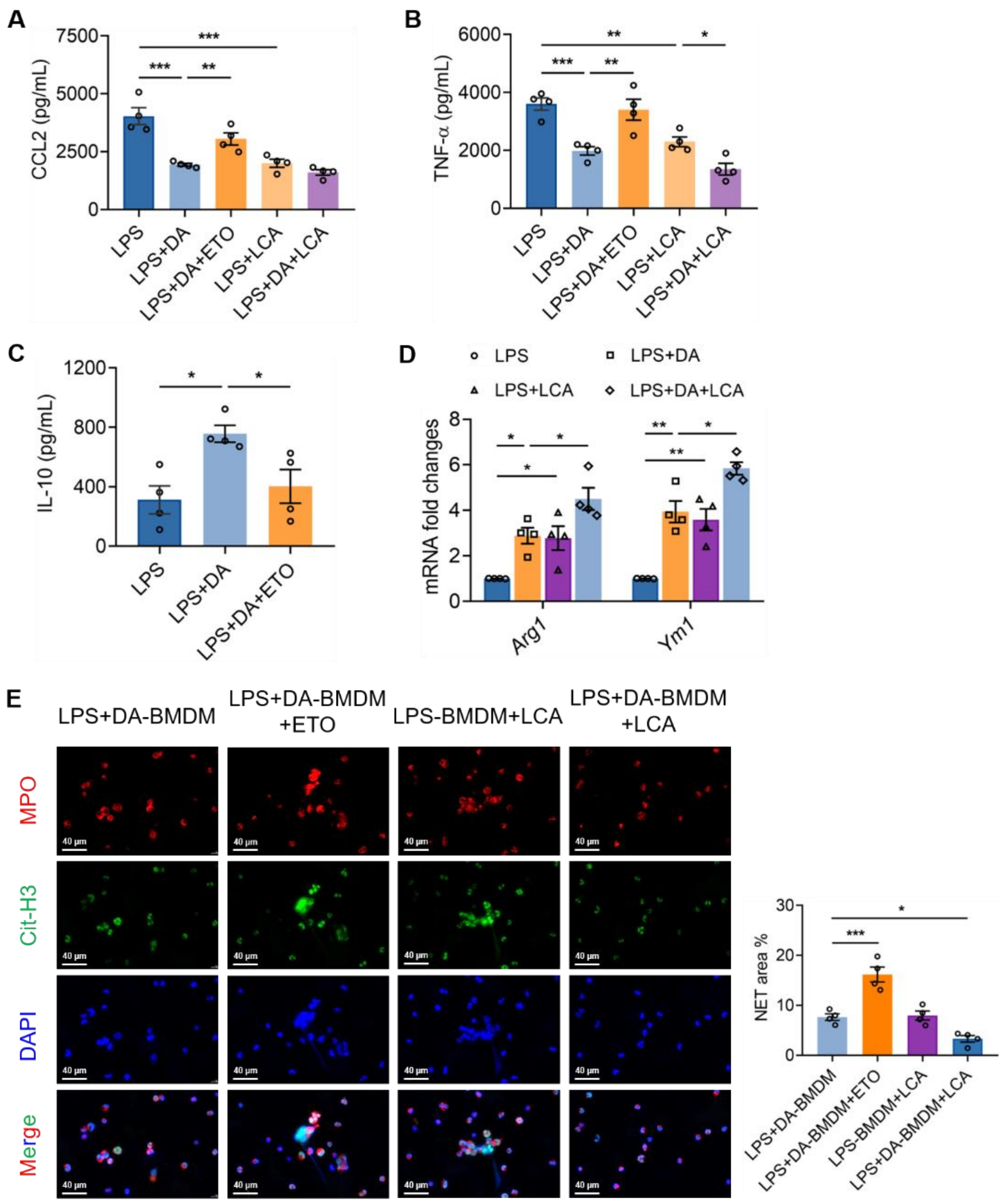

Supplementary Figure 8. (A-C) Secretions of cytokines CCL2 (A), TNF-α (B) and IL-10 (C) in BMDMs treated with DA, etomoxir (ETO), L-carnitine (LCA) or LPS (n = 4 per group). **(D)** The mRNA expressions of M2 markers *Arg1* and *Ym1* in BMDMs under indicated treatments (n = 4 per group). **(E)** Representative immunofluorescence images and the quantification of NETosis in BMDNs upon distinct treatments (n = 4 per group). NET-positive area fraction = $MPO^{+}Cit\text{-}H3^{+}$ area/total DAPI-stained nuclear area. Nuclei were stained with DAPI, displayed in blue. Scale bar: 40 μm. Data are presented as mean ± SEM and analyzed with a 95% confidence interval. Statistical

analysis was performed using one-way ANOVA followed by Bonferroni's post hoc test. *$p < 0.05$, **$p < 0.01$, ***$p < 0.001$. "n" represents the number of independent biological replicates.

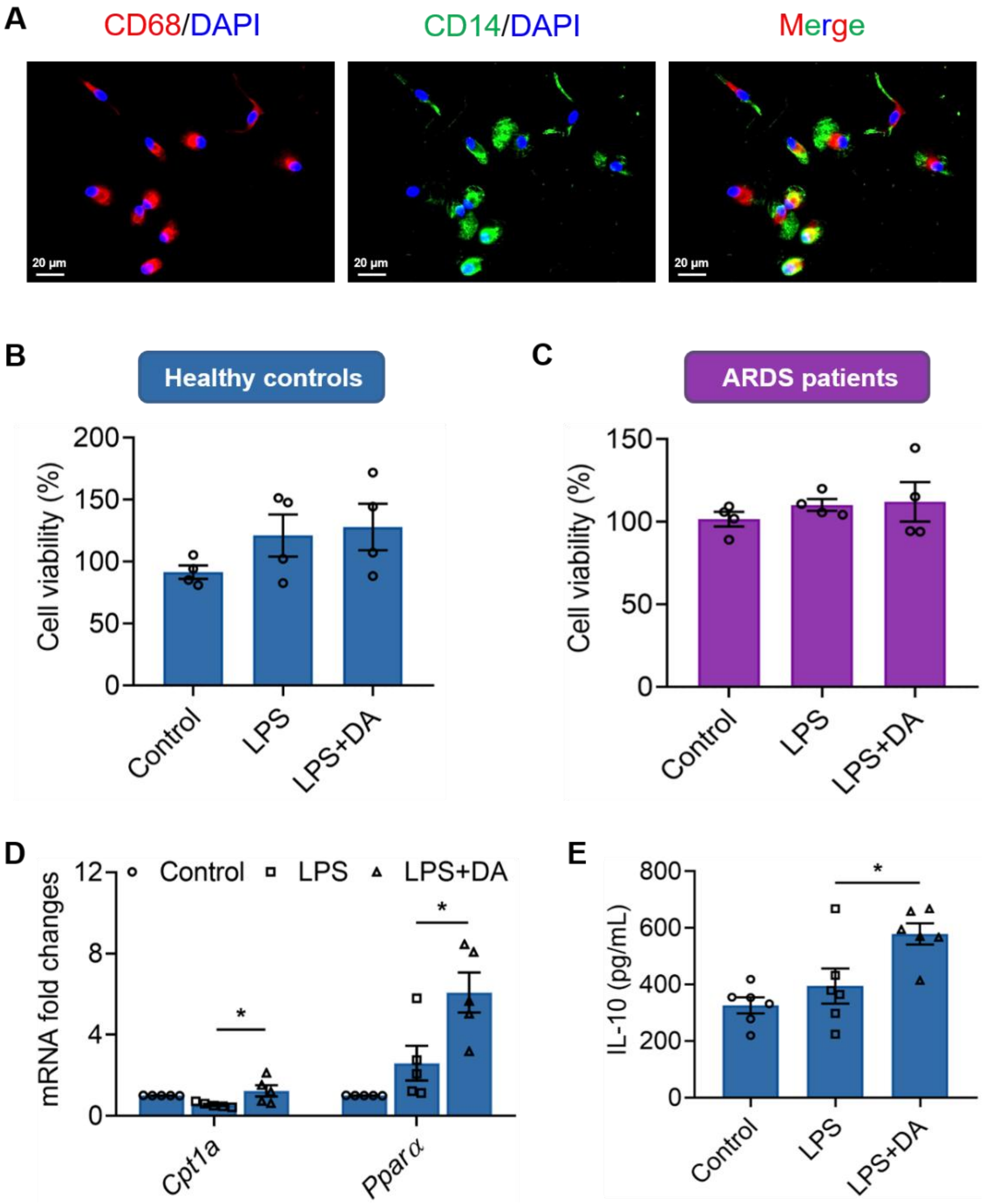

Supplementary Figure 9. (A) Immunofluorescence staining of CD68 (shown in red) and CD14 (shown in green) in human peripheral blood mononuclear cells (PBMCs)-derived macrophages. Nuclei were stained with DAPI, displayed in blue. Scale bar: 20 μm. **(B, C)** The viability of PBMCs-derived macrophages isolated from healthy donors (B) or ARDS patients (C) following indicated treatments (n = 4 per group). **(D)** The mRNA expressions of *Cpt1a* and *Ppara* in PBMCs-derived macrophages treated with DA and/or LPS (n = 5 per group). **(E)** Secretion of IL-10 in PBMCs-derived

macrophages treated with DA and/or LPS (n = 6 per group). Data are presented as mean ± SEM and analyzed with a 95% confidence interval. Statistical analysis was performed using one-way ANOVA followed by Bonferroni's post hoc test. *$p < 0.05$. "n" represents the number of independent biological replicates.

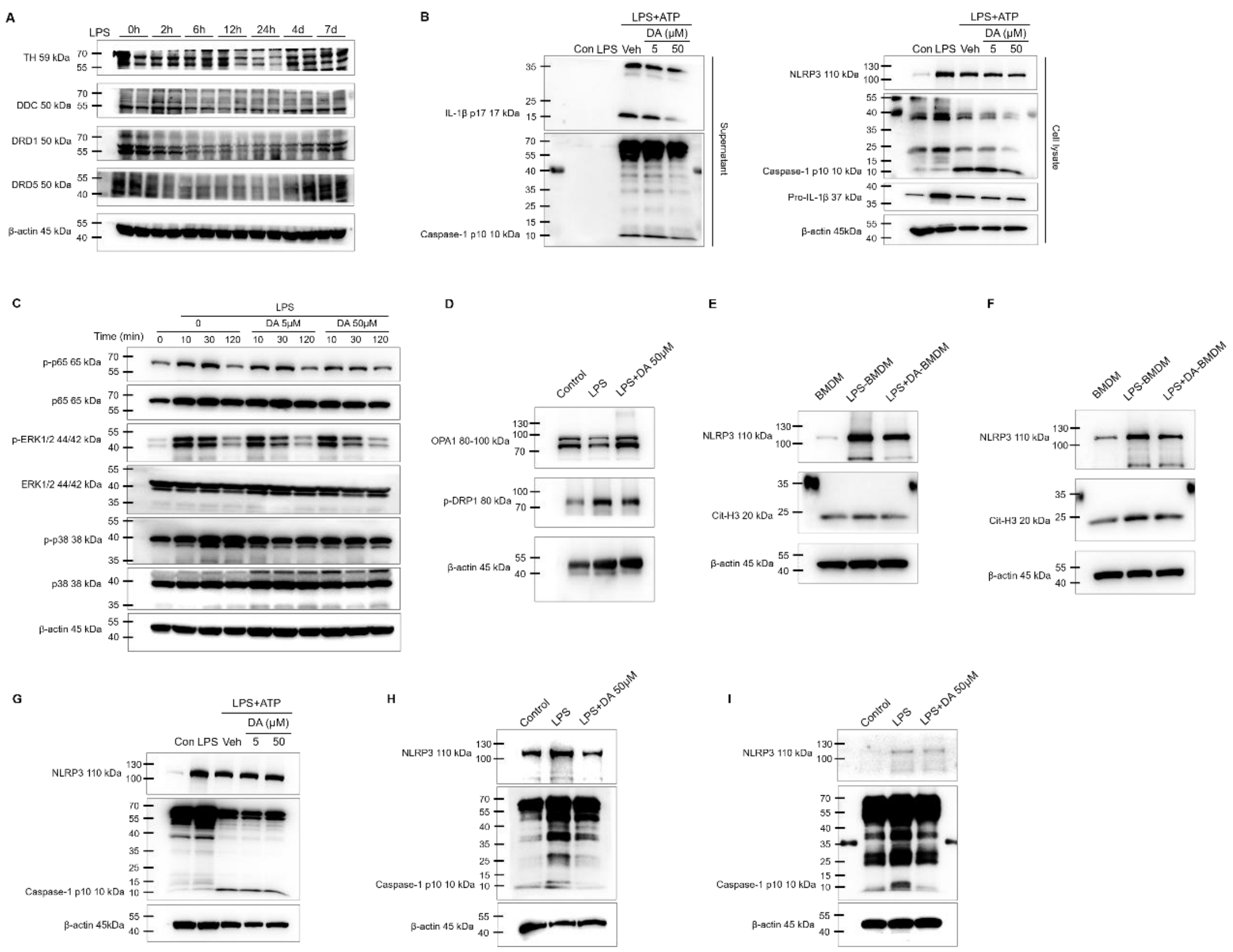

Supplementary Figure 10. (A-I) The uncropped immunoblot images corresponding to Figure 1F **(A)**, Figure 4F, J **(B, C)**, Figure 5B **(D)**, Figure 6F, J **(E, F)**, Figure 8C **(G)**, and Figure 9C, E **(H, I)** in the original text.

Supplementary Table 2. Clinical characteristics of ARDS patients with or without clinical usage of DA hydrochloride.

	**DA-naïve patients** (n=60)	**DA-treated patients** (n=30)	**X^2/Z**	***p* value**
**Age** ($\bar{x}$± *s*, year)	73.5 (61-82)	72 (64-79)	Z=-0.244	*0.807*
**Sex**			X^2=0.028	*0.868*
Female	17	8		
Male	43	22		
PaO_2	68.4 (58.1-87.8)	84.2 (65.9-101.1)	Z=-1.614	*0.107*
$PaCO_2$	38.5 (30.2-43.7)	42.6 (34.9-45.5)	Z=-1.725	*0.085*
**Cause of ARDS**				
Pneumonia	22	8	*X^2=0.900*	*0.343*
Sepsis	12	5	*X^2=0.145*	*0.703*
Abdominal diseases	13	10	*X^2=1.431*	*0.232*
Trauma	5	1	*X^2=0.201*	*0.654*
Others	8	6	*X^2=0.264*	*0.607*
**Hypertension**	20	11	*X^2=0.098*	*0.754*
**Diabetes**	13	6	*X^2=0.033*	*0.855*
**Heart diseases**	18	8	*X^2=0.108*	*0.742*
**Death**	19	13	*X^2=1.188*	*0.276*

Data are presented as median (interquartile range) for continuous variables and counts for categorical variables. Statistical analysis was performed using the chi-square (χ^2)

test for categorical variables, and the rank-sum test (presented as Z value) for continuous variables, with a 95% confidence interval for relevant analyses.

Supplementary Table 3. Demographic and clinical characteristics of 6 ARDS patients and 6 healthy volunteers.

**Group**	**ARDS**						**Healthy Control**					
**Case**	**Case 1**	**Case 2**	**Case 3**	**Case 4**	**Case 5**	**Case 6**	**Case 7**	**Case 8**	**Case 9**	**Case 10**	**Case 11**	**Case 12**
**Age (years)**	71	64	76	36	50	80	39	45	42	38	29	33
**Sex**	M	M	M	F	F	M	F	M	M	F	M	F
**Smoking**	√	×	×	×	X	√	×	×	×	×	X	X
**Pack-years, Yrs**	55	-	-	-	-	60	-	-	-	-	-	-
**Cancer history**	×	√	×	×	X	√	×	×	×	×	X	X
**Sample**	Peripheral blood											
**Underlying diseases**												
Hypertension	√	×	√	×	X	√	×	×	×	×	X	X
Diabetes	×	×	×	×	X	×	×	×	×	×	X	X
Cardiac disease	√	×	×	×	X	√	×	×	×	×	X	X
Autoimmune diseases	×	×	×	×	X	×	×	×	×	×	X	X
Others	×	×	×	×	X	×	×	×	×	×	X	X
**Clinical profile**												
Disease severity[*]	Severe	Moderate	Moderate	Moderate	Mild	Severe	NA	NA	NA	NA	NA	NA
Treatment background[#]	A + B + C + D + E	A + B + C + D + E	A + B + C + D	B + C + D	A + B + D	A + B + C + D + E	NA	NA	NA	NA	NA	NA

Outcome: Survival	×	×	×	√	√	×	√	√	√	√	√	√

*ARDS disease severity was defined according to the Berlin criteria, based on the PaO_2/FiO_2 ratio with PEEP≥5 cmH_2O, mild ($200 < PaO_2/FiO_2 \leq 300$ mmHg), moderate ($100 < PaO_2/FiO_2 \leq 200$ mmHg), and severe ($PaO_2/FiO_2 \leq 100$ mmHg).

#Treatment modalities were categorized as follows: A, antibiotics; B, systemic corticosteroids; C, circulatory support (including vasopressors and fluid management); D, conventional respiratory support (including invasive mechanical ventilation with lung-protective strategies); E, advanced respiratory support (including prone positioning and extracorporeal membrane oxygenation).
